# Development of visual-stimulus reversal learning-memory in mice is dependent on social interaction

**DOI:** 10.1016/j.isci.2026.114864

**Published:** 2026-01-31

**Authors:** Sarah Wicki, Annika Canziani, Giulia Poggi, Ali Özgür Argunşah, Theofanis Karayannis, Christopher R. Pryce

**Affiliations:** 1 Preclinical Laboratory for Translational Research into Affective Disorders, University Hospital of Psychiatry and University of Zurich, Zurich, Switzerland; 2 Laboratory for Neural Circuit Assembly, Brain Research Institute, University of Zurich, Zurich, Switzerland; 3 University Research Priority Program (URPP), Adaptive Brain Circuits in Development and Learning (AdaBD), University of Zurich, Zurich, Switzerland; 4 Neuroscience Center Zurich, University of Zurich and ETH Zurich, Zurich, Switzerland

**Keywords:** Biological sciences, Neuroscience, Sensory neuroscience

## Abstract

Flexible learning about visual stimuli and reward was investigated in male mice in terms of development, social stimulation, and memory. In a sucrose foraging task, mice learned that one of the two complex visual stimuli was correct (compound discrimination, CD), followed 1 day later by rule reversal (CD reversal, CDR). Socially reared (SR) mice aged 6–12 weeks were tested at one weekly age: while all ages had CDR > CD errors, CDR errors were low at week 6, possibly due to immature long-term memory. Next, effects of social condition and memory were investigated: SR and socially isolated (SI, weeks 5–8) mice were tested on same day CD-CDR (working memory) or consecutive day CD-CDR (long-term memory): in each condition, SR mice had CDR > CD errors; SI mice had this in working memory, but CDR = CD errors in long-term memory, possibly reflecting easier reversal due to the prolongation of immature long-term memory.

## Highlights

• Social mice display fewer long-term reversal-learning errors at week 6 vs. 7
• Isolated vs. social mice (week 5–8) display typical short-term reversal-learning
• Isolated vs. social mice (week 5–8) display fewer long-term reversal-learning errors
• Social isolation retains long-term memory in a less-efficient adolescent state

## Graphical abstract

**Figure:**
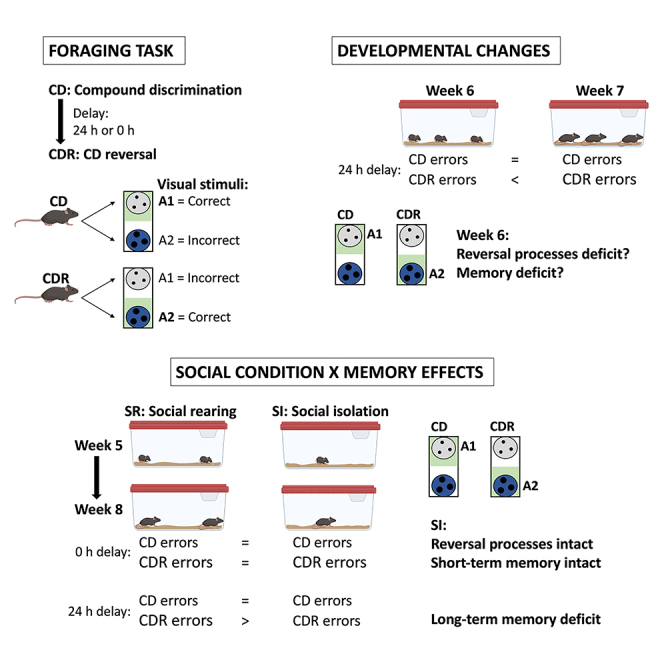

## Introduction

Executive functioning refers collectively to diverse neuropsychological processes that enable the learning and monitoring of changing associations between discriminative stimuli, behavior, and emotionally salient stimuli.^1^^,^^2^ Various tasks have been established to enable the study and measurement of executive functions, and notable among these are the intra-dimensional/extra-dimensional (ID/ED) attentional set-shifting tasks that are applied in different species.^3^^,^^4^ Human ID/ED tasks typically involve the presentation on a touch-sensitive computer screen of visual stimuli from two different “sets,” such as white lines and blue shapes. At the so-called compound discrimination (CD) stage, two exemplars of each set are presented, typically with 1 exemplar from each of the 2 sets superimposed on each other in a randomized manner across trials, and responding to one exemplar of the attentional set is associated with positive reinforcement. The ID and ED set-shifts refer to presentations of pairs of new stimuli from the same sets, to enable the assessment of the subject’s ability to continue attending to the same set in the case of an ID shift (IDS) and to re-direct attention to the opposite set in the case of an ED shift (EDS).^3^ In addition, rule reversals can be introduced to enable the assessment of flexibility in terms of overcoming perseveration, i.e., inhibiting responding to the previously correct stimulus, and overcoming learned non-reward, i.e., discontinuing avoidance of the previously incorrect stimulus.^5^^,^^6^ Whilst such human-computer screen tasks can also be applied in monkey studies,^7^^,^^8^^,^^9^ and indeed in rodent studies,^10^^,^^11^ in rats and mice, a three-dimensional foraging version of the ID/ED task has proven particularly effective and species appropriate.^12^^,^^13^^,^^14^ Typically using two foraging bowls and a sucrose pellet buried in one of the bowls as a reward, stimulus sets used can include different somatosensory substrates as digging media, colored (visual) beads as digging media, odors applied to the digging medium, and somatosensory textures applied to the foraging bowls. The ID/ED tasks, whether in primate or rodent, are typically presented with the different stages in a specific order and as a continuous sequence within one daily session.^8^^,^^12^^,^^15^ Consequently, reversal learning stages, and IDS and EDS, follow their predecessor stages immediately, and it is the working memory of the previous stage that needs to be overcome in terms of perseveration, learned non-reward, and maintaining or redirecting attention.

One majorly important aspect of executive functioning is its development.^16^ The study of the development of executive functions is challenging, given that it requires identifying tasks that can be applied across a wide range of life stages, such as from juvenility to adulthood. In general terms, processes emerge within species-specific (st)age-dependent critical periods.^17^^,^^18^ These will be determined in part by the maturational state of the prefrontal cortex (PFC); the PFC is the major brain region within the neural circuitry underlying executive functions,^2^^,^^19^ and it is characterized by marked postnatal maturation,^20^^,^^21^^,^^22^ including undergoing the process of synaptic pruning in adolescence-young adulthood.^19^ A major contributor to such pruning is glutamate receptor-mediated synaptic plasticity.^23^ Maturation of the PFC, and therefore of psychological processes dependent on it, is also dependent on species-typical stimulatory inputs from the environment, with social interactions and relationships constituting one of the most important sources of such input.^24^^,^^25^ The development of executive functions has been investigated in a small number of studies in rats or mice to date. In rats, using a foraging ID/ED task with several stages within one daily session, adolescents were less proficient than adults at the CD and EDS stages and at the reversal stages.^26^ An ID/ED study in mice comparing adolescents and adults used either 4-choice or 2-choice odor discrimination tasks: adolescents displayed improved reversal learning relative to adults in the 4-choice task.^27^ Another ID/ED study in mice comparing adolescents and adults used an automated operant apparatus in which subjects required many trials per stage and therefore typically completed only one stage per day; this meant that reversal learning typically took place 24 h after learning, such that long-term memory could also contribute to task performance. Under these conditions, adolescents were more proficient than adults at CD reversal and IDS reversal, possibly due to immaturity of long-term memory and therefore easier reversal.^11^ Concerning environmental inputs to the development of executive functioning, in rats, studies have investigated the effects of social isolation (SI) from weaning until early adulthood on subsequent foraging task behavior. In one study in which all stages were tested, SI female rats were impaired at ED set-shifts specifically, with no effect on reversal learning^28^; in two studies in which CD reversal was the most complex stage tested, SI rats were impaired at CD reversal learning.^29^^,^^30^ Mouse studies of SI effects on cortical structure-function report decreased spike activity in PFC glutamate pyramidal neurons,^31^ decreased connectivity of the orbital (prefrontal) cortex (OC),^32^ and altered status of OFC glutamate neuron synapses at basolateral amygdala neurons.^33^ In rats, SI led to decreased dendritic spine density of mPFC glutamate pyramidal neurons.^34^

With regards to cortical involvement in set-shifting and reversal learning, adult rodent excitotoxic lesion studies provide evidence for specific structure-function associations. Therefore, in rats and mice, lesioning of the medial PFC (prelimbic and infralimbic cortices) led to impaired ED set-shifting and was without effect on reversal learning at any stage of the foraging ID/ED task.^12^^,^^35^ Also in rats and mice, excitotoxic lesioning of the orbital (prefrontal) cortex (OC), focusing on the ventral, medial, and lateral sub-regions, led to impaired reversal learning at each stage of the ID/ED task, while being without effect on ID or ED set-shifting.^15^^,^^35^ The reversal learning deficit induced by OC lesioning was underlain by difficulty in overcoming the influence of learned non-reward on stimulus choice.^5^ Lesions of the basal forebrain also impaired reversal learning without impacting the shifting of attentional sets.^36^ Major among the neuronal populations organized in a layered manner in the OC are pyramidal glutamatergic neurons that project to and synapse with other glutamatergic neurons and to GABAergic neurons,^37^ and it is likely that excitotoxic lesioning of these neurons and their synapses mediated the observed effects on executive functions.

Against the above background, in the current mouse study, we firstly investigated the effects of adolescence-to-adulthood development on the stages, simple discrimination, CD, CD reversal, and IDS, of the ID/ED foraging task, using visual stimuli as reward and somatosensory stimuli as distractor. Visual stimuli were used to increase the translational relevance of the paradigm to human and monkey studies. We modified the task by presenting only one task stage per day, to minimize the effects of satiety and fatigue, and also to increase the relevance of long-term (24-h) memory to the effects of each task stage on the subsequent stage. We identified an effect of development on CD reversal in terms of adolescent mice reversing the stimulus-reward rule more readily. Second, we investigated the effects of adolescent social isolation on task behavior: given that the effect of development on CD reversal could be attributable to perseveration, learned non-reward, or long-term memory, we designed one experiment to facilitate differentiation between these processes; testing was conducted for CD and CDR on the same day or on successive days, such that they would be sensitive to detecting SI effects on working memory and long-term memory, respectively. Social isolation resulted in mice making relatively few CD reversal errors in the long-term memory condition specifically, suggesting that this type of memory was impaired, and that SI had suspended its development at the adolescent stage. Given that the effects of both development and SI were on CD reversal, at the neuroanatomical level, we investigated their effects on orbital cortex specifically, this being the PFC region implicated in reversal learning; the presence of consistent but small effects on synaptic protein levels suggests that other brain regions were more substantively responsible for these behavioral effects.

## Results

### Experiment 1: Species-typical development of complex sensory learning

**Figure 1:**
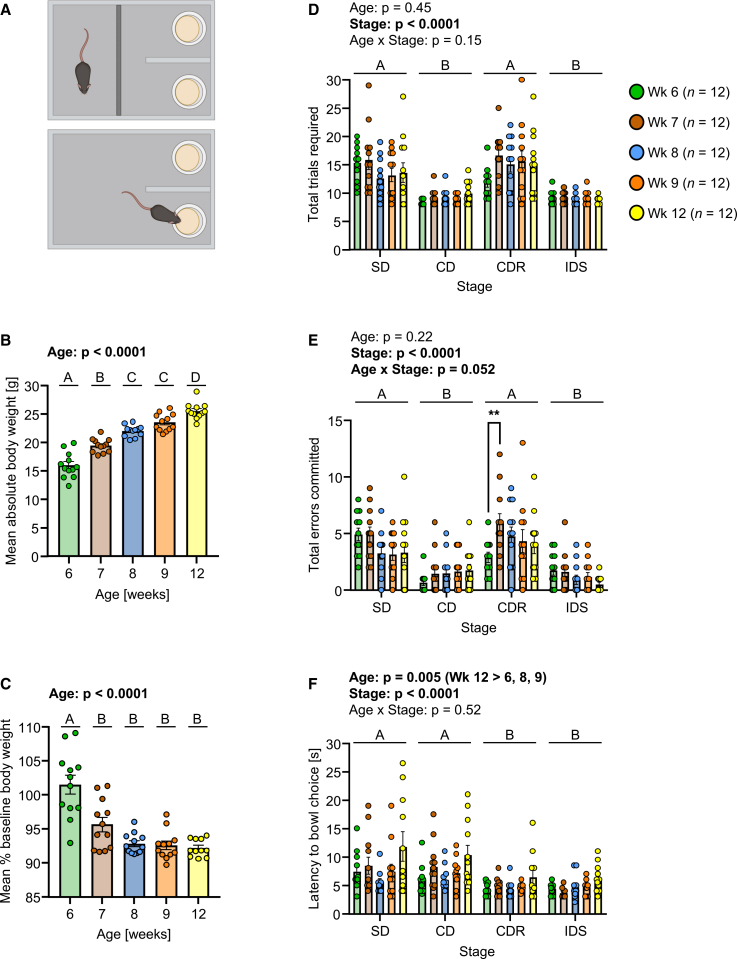
Effects of age on behavior in the complex sensory learning task testing 1 stage per day (A) Schematic of the CSL task apparatus, showing the mouse in the starting compartment during the waiting phase (upper) and in the stimulus compartments during the bowl choice phase (lower). (B) Mean absolute body weight per mouse across the 4 days of testing. (C) Mean body weight per mouse as a percentage of baseline, where the latter was obtained immediately prior to the onset of food restriction for training and testing. In (B and C), statistical analysis was conducted using one-way ANOVA with between-subject factor of age. In the case of a significant age effect, post hoc pairwise comparisons were conducted using Tukey’s multiple comparisons test: ages denoted by different letters are significantly different from one another. (D) Total trials required to learning criterion according to age and stage. (E) Total errors committed to learning criterion according to age and stage. (F) Median latency to bowl choice according to age and stage. In (D–F), statistical analysis was conducted using linear mixed-effect models with fixed effects of age and stage and a random effect of mouse ID. In the case of significant main effect of stage, post hoc pairwise comparisons were conducted using Tukey’s test: stages denoted by different letters, e.g., A vs. B, A vs. C, B vs. C, were significantly different from each other at *p* < 0.05 or lower, whereas stages denoted by the same letter, e.g., A vs. A, were not significantly different from each other. In the case of a significant age × stage interaction effect, post hoc pairwise comparisons were conducted using Tukey’s test: ∗∗*p* < 0.01. In (B–F), the data are individual values and group mean ± SEM. In all cases, outliers were identified using the ROUT method (Q = 1%) and removed: (B) no outliers; (C) 0–1 per age group, total = 1/60; (D) 0–3 per age x stage: total = 14/240; (E) 0–1 per age x stage: total = 3/240; (F) 0–3 per age x stage: total = 10/240. Images in (A) were created with BioRender.com.

The aims of Expt. 1 were to investigate species-typical development of complex sensory learning (CSL) and maturation of orbital cortex (OC) from adolescence to adulthood. Training and testing in the CSL task (CSLT) were conducted under mild food restriction in a 2-week period per mouse, at ages 5–6, 6–7, 7–8, 8–9 or 11–12 weeks (*n* = 12 per age group) (Figure S1). Each of the CSLT training and testing had a maximum duration of 5 days, Monday-Friday, in consecutive weeks. Therefore, in the first week, training was conducted and comprised 4 stages of digging in stimulus bowls containing gradually increasing amounts of sawdust to retrieve sucrose pellets. Mice could pass 1 or 2 stages per session and day; however, if mice were progressing relatively slowly, they were given two training sessions, several hours apart, on the same day. For further details, see STAR Methods. In the second week, mice proceeded to testing, in which they were presented with 2 lemon odour-treated bowls containing beads, one specific color-shade per bowl (i.e., visual discriminatory stimulus dimension), and wrapped with textured material, one specific texture per bowl (i.e., somatosensory distractor stimulus dimension). The bowls were placed in a CSLT apparatus (Figure 1A), one per stimulus compartment and out of sight of the mouse in a starting compartment. At each testing trial, a sucrose pellet was buried in one of the bowls, and the mouse needed to acquire the predictive association that existed between one exemplar of the relevant sensory stimulus dimension (i.e., visual) and sucrose reinforcement. For all mice, the visual-somatosensory stimulus combination and left-right positioning of the bowls across trials followed a pre-set and pseudo-randomized schedule. Testing comprised 4 stages. Stage 1, simple discrimination (SD): one bowl contained white beads, and the other dark-blue beads, and both bowls had a smooth, ceramic texture with no somatosensory stimuli applied. One color-shade was reinforced, and this was counterbalanced across mice within each group. Stage 2, compound discrimination (CD): two bowls were covered with fine sandpaper and contained either white beads or dark-blue beads, and two with coarse sandpaper and contained either white or dark-blue beads. Per trial, 1 fine and 1 coarse bowl were presented, and 1 contained white and 1 dark-blue beads, and the color-shade reinforced at SD was relevant and texture was irrelevant, i.e., formation of an attentional set “visual stimulus” was reinforced. Stage 3, CD reversal (CDR): the previously incorrect color-shade was now correct and vice versa, while texture remained present and irrelevant. Stage 4, intra-dimensional shift (IDS): two novel bead color-shades were introduced - light-blue and brown - as were two new textures - ridged versus smooth cardboard. Attentional set visual stimulus was still correct, and the ability to apply this to novel stimuli was tested. Bowl choice was defined as displaying digging behavior, i.e., mouse moving beads in the bowl with its front paws and/or snout; only contacting or sniffing the bowl, and/or nibbling on a texture, was not a bowl choice. Each daily testing session had a maximum of 30 trials per stage, and a maximum duration of 60 min. Learning criterion for passing a stage was 8 correct choices in 8–10 consecutive trials. If a mouse did not pass a stage within the 30 trials, it began the next day’s session on this same stage, and the number of consecutive correct trials was reset to 0. Testing was completed when all 4 test stages were passed.

Mice were mildly food restricted to 90–95% BBW or 95–100% BBW in the case of week 5–6 mice (to allow continued growth), to ensure that they were motivated for sucrose pellet reward. As expected, during the 1-week testing period, absolute BW increased with age (group main effect: *F*_*4,55*_ = 70.96, *p* < 0.0001; Figure 1B). For % BBW during the testing period, there was a group main effect (*F*_*4,54*_ = 19.78, *p* < 0.0001; Figure 1C): post hoc testing demonstrated that % BBW was higher in mice aged 6 weeks than in older mice, as planned; thereafter BBW was consistently at 90–95%.

In CSLT training, for the total number of sessions required, there was a group main effect (*F*_*4,55*_ = 3.45, *p* = 0.014): post hoc analysis revealed that mice aged 12 weeks needed more training sessions (7.3 ± 0.65, mean ± SEM) than mice aged 7 weeks (4.4 ± 0.45) or 8 weeks (4.8 ± 0.64). In CSLT testing, the total number of trials required (Figure 1D) differed according to stage (stage main effect: *F*_*3,151*_ = 49.48, *p* < 0.0001), with mice requiring fewer trials at CD than SD, more trials at CDR than CD, and fewer trials at IDS than SD and CDR. There was no main effect of age (*F*_*4,55*_ = 0.94, *p* = 0.45) or interaction effect of age x stage. For total errors committed to reach the learning criterion (Figure 1E), there was a main effect of stage (*F*_*3,162*_ = 43.55, *p* < 0.0001), with post hoc analysis revealing the same pairwise differences as for total trials required. Furthermore, there was an age × stage interaction effect (F_12,162_ = 1.80, *p* = 0.052): despite the *p*-value being slightly higher than α, exploratory post hoc analysis was conducted and revealed that, at CDR specifically, mice aged 6 weeks committed fewer errors than mice aged 7 weeks (predicted LS-mean difference = −2.98, 95% confidence interval = −5.36 to −0.61, *p* = 0.006, Tukey’s multiple comparisons test). For all other pairwise age-group comparisons for all stages, the 95% confidence interval for the difference in the means contained zero, with *p* ≥ 0.16 in Tukey’s multiple comparisons test. For the latency to bowl choice (Figure 1F), there was a main effect of stage (*F*_*3,155*_ = 19.27, *p* < 0.0001), with mice having shorter latencies at CDR and IDS than at SD and CD. There was a main effect of age (*F*_*4,55*_ = 4.18, *p* = 0.005), with mice aged 12 weeks requiring more time to make a bowl choice than mice aged 6, 8, or 9 weeks. Given that mice aged week 6 were, according to their % baseline body weight at testing, the least food restricted, and mice aged week 7 were the next least food restricted (Figure 1C), differences in food restriction and therefore hunger/motivation do not account for the increase in CDR errors between ages 6 weeks and 7 weeks.

### Species-typical maturation of orbital cortex glutamatergic synaptic proteins

**Figure 2:**
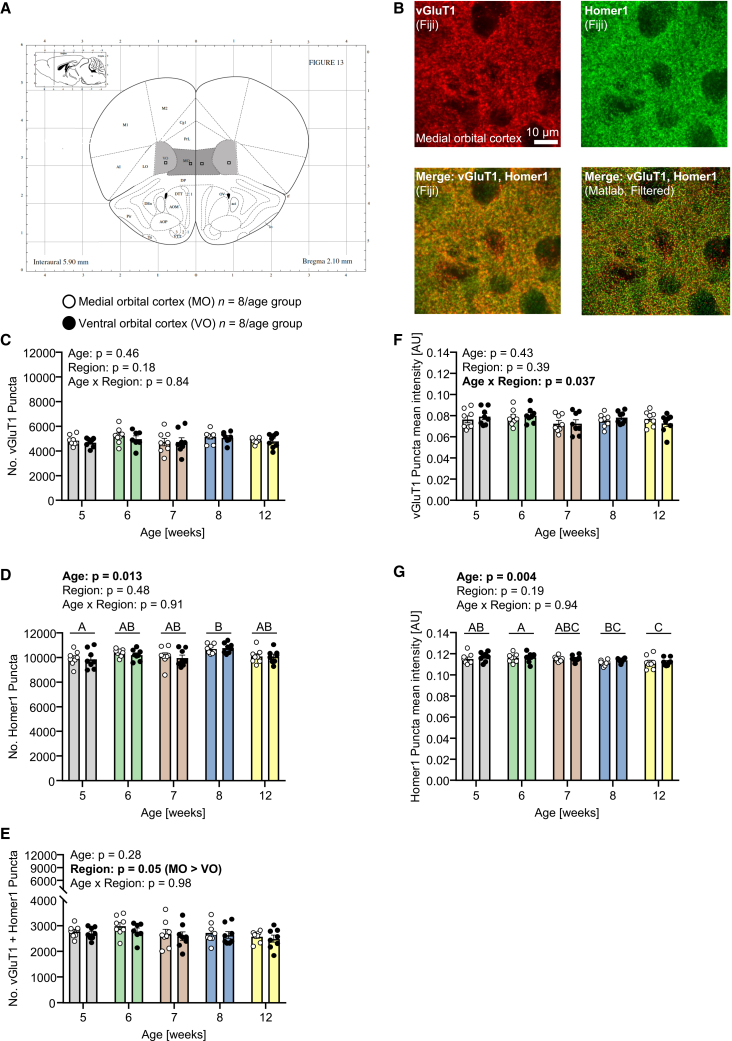
Effects of age on maturation of orbital cortex glutamate synaptic proteins (A) Coronal section image (bregma +2.10 mm), from the Mouse Brain Atlas (Paxinos & Franklin, 2007), containing the orbital cortex (OC) ROIs: medial and ventral OC (MO, VO). The squares indicate the locations and the sizes of the ROIs at image acquisition. (B) Representative micrographs of MO showing immunofluorescence co-staining for vesicular glutamate transporter 1 (vGluT1), used as pre-synaptic marker, and post-synaptic density scaffolding protein (Homer1), used as post-synaptic marker. Micrographs: vGluT1 (red), Homer1 (green), merged vGluT1 and Homer1 (yellow). Scale bars, 10 μm. Brightness and contrast have been adjusted for display purposes. The lower-right image displays the visual output of the MATLAB script used for synaptic puncta detection and colocalization analysis for the images shown. The micrographs for VO were similar. (C) Number of vGluT1 puncta according to age and OC subregion. (D) Number of Homer1 puncta according to age and OC subregion. (E) Number of colocalized vGluT1 + Homer1 puncta according to age and OC subregion. In (C–E), statistical analysis was conducted using either two-way ANOVA or, in the case of missing within-subject values, linear mixed-effect models. In the case of a significant main effect of age, post hoc pairwise comparisons were conducted using Sidak’s test: ages denoted by different letters, i.e., A vs. B, were significantly different from each other at *p* < 0.05 or lower, whereas ages denoted by the same letters, i.e., A vs. AB, B vs. AB, were not significantly different from each other. (F) vGluT1 puncta mean intensity according to age and OC subregion. (G) Homer1 puncta mean intensity according to age and OC subregion. In (F and G), statistical analysis was conducted using two-way ANOVA. In (F) there was a significant age x OC subregion interaction effect but post hoc testing was non-significant. In (G) there was a significant main effect of age: post hoc pairwise comparisons were conducted using Tukey’s test, and ages denoted by different letters, i.e., A vs. BC, A vs. C, were significantly different from each other at *p* < 0.05 or lower, whereas ages denoted by the same letters, e.g., A vs. AB, ABC vs. BC, were not significantly different from each other. In (C–G), the data are individual values and group mean ± SEM. In all cases, outliers were identified using the ROUT method (Q = 1%) and removed: (C) 0–1 per age x region: total = 1/80; (D) no outliers; (E) 0–1 per age x region: total = 1/80; (F) no outliers; (G) no outliers.

Given the above evidence for developmental changes in reversal learning-memory, we focused our investigation on maturational changes in synaptic proteins in the orbital cortex (OC), which, as described in the Introduction, is essential to the re-learning of stimulus-reward associations in complex sensory reversal learning in ID/ED tasks. Vesicular glutamate transporter 1 (vGluT1) was used as a pre-synaptic protein, and post-synaptic density scaffolding protein (Homer1) as post-synaptic protein. Co-localization of these two proteins is an indicator for an excitatory glutamate synapse. In naive same-sex littermates of the mice studied behaviorally, species-typical maturation of OC glutamate synapses was studied in terms of immunofluorescence staining, using mice aged 5, 6, 7, 8, or 12 weeks (*n* = 8 per age group; the age 9 weeks was not studied). The experiences of CSL training and testing, and inter-individual differences therein, could impact the state of synaptic proteins, and differentially so, and hence the neuroanatomical experiments were conducted in the naive littermates of the mice studied behaviorally, and at the appropriate maturational age. The littermates of the mice that underwent CSLT testing were euthanized three days after testing completion, so that brain maturation status corresponded closely to that of the littermate during behavioral testing. These naive neuroanatomy littermates underwent perfusion-fixation, and coronal brain sections (40 μm) were cut. Per mouse, two sections (bregma level +2.10/2.06 mm) containing the ROIs medial OC (MO) and ventral OC (VO) were used for immunofluorescence co-staining for vGluT1 and Homer1. A dedicated MATLAB script was used for synaptic puncta detection and colocalization analysis (Figures 2A and 2B). For vGluT1 and Homer1, the size and shape of immunofluorescence signals were used to define the presence of puncta, and the average intensity of these signals provided a measure of the amount of protein signal at each punctum. For vGluT1 puncta, there was no effect of age or OC subregion (Figure 2C). For Homer1 puncta, there was a main effect of age (*F*_*4,35*_ = 3.7, *p* = 0.013; Figure 2D), with mice aged 5 weeks having fewer than mice aged 8 weeks (mean difference = −831, 95% CI = −1544 to −119, *p* = 0.01), with similar levels in the 2 OC subregions. For synaptic puncta, these were similar across ages, and there were consistently more in MO than VO (main effect of region: *F*_*1,34*_ = 4.13, *p* = 0.05; Figure 2E). Applying the same statistical models to analyze mean puncta intensity, for vGluT1, there was an age x OC subregion interaction effect (*F*_*4,35*_ = 2.88, *p* = 0.037; Figure 2F), but the tendencies for MO > VO mean puncta intensity at ages weeks 5–7 were not significant. For Homer1, there was a main effect of age (*F*_*4,35*_ = 4.7, *p* = 0.004; Figure 2G), with mice aged 5 weeks and in particular 6 weeks having higher mean puncta intensity than older mice.

Therefore, we were able to establish a modified version of the rodent attentional-set foraging task that is restricted to the stages SD, CD, CDR and IDS, but delivers the advantages that it can be conducted in mice as young as 5–6 weeks, training and testing can be completed within a 2-week period while testing one stage per day, and the attentional set comprises visual stimuli as is the case in the analogous primate, including human, tasks. Using this CSLT, mice aged 6 weeks committed fewer errors at CDR than did mice aged 7 weeks, suggesting that changes in neurobehavioral processes underlying reversal learning, such as perseveration or learned non-reward, or in long-term memory, take place during this developmental period. The OC is known to be essential for efficient reversal learning, and immunostaining for synaptic proteins identified that mice aged 5 weeks had fewer post-synaptic Homer1 puncta than did mice at 8 weeks, and that the amount of Homer1 at each punctum was higher in mice aged 5 and 6 weeks than in adult mice.

### Experiment 2: Effects of adolescent social isolation on complex sensory learning

A major aim of this study was to investigate the importance of social interaction, specifically, the impact of its deprivation by social isolation, on the development of complex sensory learning. Based on the evidence from Expt. 1 that mice aged 6 weeks committed fewer CD reversal errors than mice aged 7 weeks (Figure 1E), possibly reflecting the emergence of processes that regulate reversal learning across this developmental window, e.g., perseveration, learned non-reward, and memory, we investigated the effects of social isolation (SI) across this period on CSLT behavior. From week 5 (27–33 days) until the end of the experiment at age week 8, mice were maintained in one of the two social conditions: they either remained with 1, occasionally 2, male littermates, which was the control condition of social rearing (SR), or littermates were separated and socially isolated (SI) (Figure S1).

**Figure 3:**
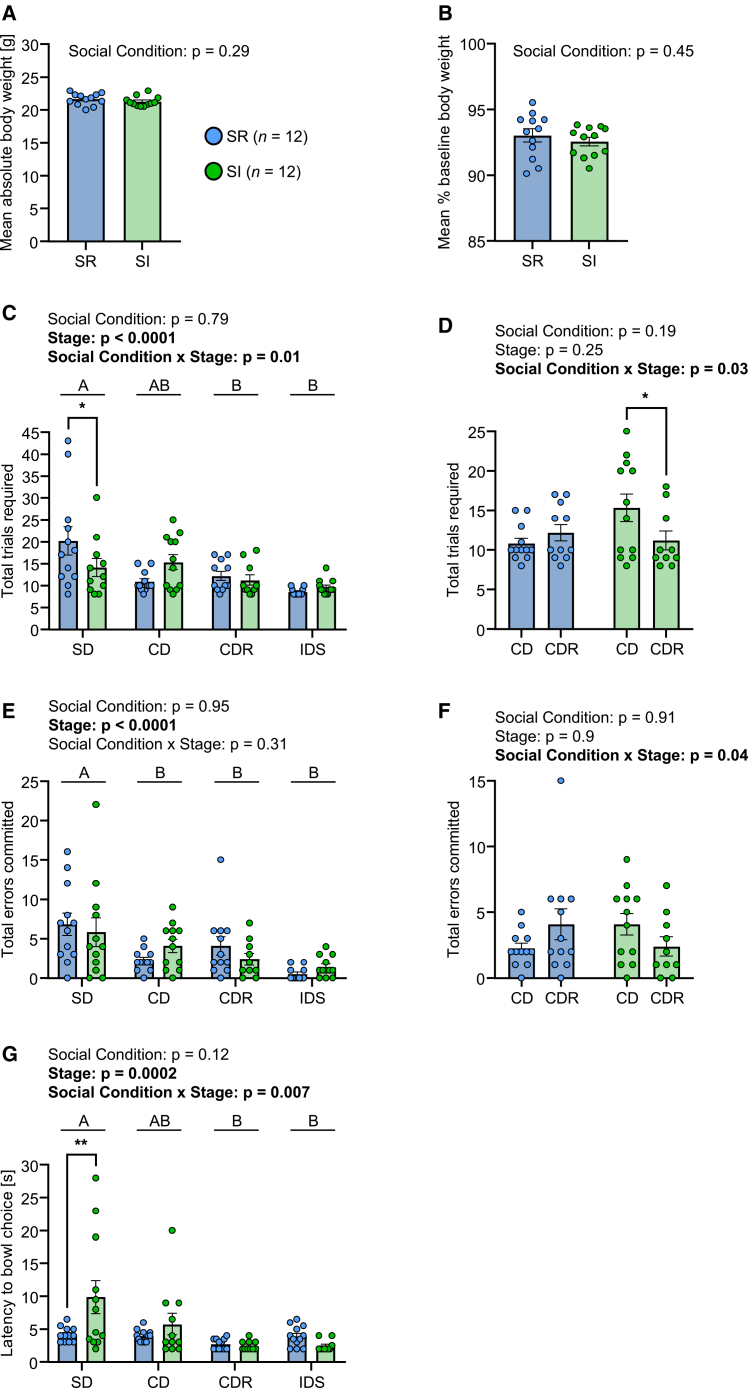
Effects of adolescent social isolation on behavior in the complex sensory learning task testing 1 stage per day (A) Mean absolute body weight per mouse across the 4 days of testing. (B) Mean body weight per mouse as a percentage of baseline, where the latter was obtained immediately prior to the onset of food restriction for training and testing. In (A and B), statistical analysis was conducted using a two-tailed unpaired *t* test. (C) Total trials required to learning criterion according to social condition and stage. (D) Total trials required to learning criterion for CD versus CDR stages specifically. (E) Total errors committed to learning criterion according to social condition and stage. (F) Total errors committed for CD versus CDR stages specifically. (G) Median latency to bowl choice according to social condition and stage. In (C, E and G), statistical analysis was conducted using linear mixed-effect models with fixed effects of social condition and stage and a random effect of mouse ID. In the case of a significant main effect of stage, post hoc pairwise comparisons were conducted using Tukey’s test: stages denoted by different letters, i.e., A vs. B, were significantly different from each other at *p* < 0.05 or lower, whereas stages denoted by the same letters, i.e., A vs. AB, B vs. B, were not significantly different from each other. In the case of a significant social condition × stage interaction effect, post hoc pairwise comparisons were conducted using Tukey’s test: ∗*p* < 0.05, ∗∗*p* < 0.01. In (D and F), statistical analysis was conducted using linear mixed-effect models with fixed effects of social condition and stage and a random effect of mouse ID. In the case of a significant social condition × stage interaction effect, post hoc pairwise comparisons were conducted using Sidak’s test: ∗*p* < 0.05. In (A–G), the data are individual values and group mean ± SEM. In all cases, outliers were identified using the ROUT method (Q = 1%) and removed: (A) no outliers; (B) no outliers; (C) 0–2 per social condition x stage, total = 5/96; (D) 0–2 per social condition x stage, total = 3/48; (E) 0–2 per social condition x stage: total = 3/96; (F) 0–2 per social condition x stage: total = 2/48; (G) 0–2 per social condition x stage: total = 5/96.

Socially reared and SI mice were mildly food restricted to 90–95% BBW during training and testing. For BW during testing (Figure 3A), there was no difference between SI and SR mice (two-tailed unpaired *t* test: t = 1.09, df = 22, *p* = 0.29), and this was also the case for % BBW (two-tailed unpaired *t* test: t = 0.77, df = 22, *p* = 0.45; Figure 3B), with mice maintained within the target range of 90–95% BBW. At training, SR (4.3 ± 0.62) and SI (3.6 ± 0.62) mice required a similar number of sessions to complete the 4 stages (two-tailed unpaired *t* test: t = 1.07, df = 22, *p* = 0.30). At CSLT testing, for total trials required (Figure 3C) there was a social condition × stage interaction effect (*F*_*3,61*_ = 3.93, *p* = 0.01): at SD, SI mice required fewer total trials than SR mice, and the 2 groups were similar in total trials at the other stages. Total errors committed were similar in SI and SR mice (Figure 3E); in terms of stage, SR and SI mice committed more errors at SD than at subsequent stages (stage main effect: *F*_*3,63*_ = 9.8, *p* < 0.0001). For latency to bowl choice (Figure 3G), there was a social condition × stage interaction effect (*F*_*3,61*_ = 4.43, *p* = 0.007): again, the SI effect was specific to SD, with SI mice taking longer to make a choice than SR mice, and the stage-specific latencies of the 2 groups being similar thereafter. We were particularly interested in behavior at CDR relative to CD and the effects of SI thereon; therefore, as a planned comparison, we analyzed these 2 stages specifically. For total trials required (Figure 3D) there was a social condition × stage interaction effect (*F*_*1,19*_ = 5.74, *p* = 0.03): whereas SR mice required a similar number of total trials at CDR to CD (*p* = 0.63), SI mice actually required fewer trials at CDR than CD (predicted LS-mean difference = 3.94, 95% CI = −0.11 to −7.77, *p* = 0.04 in Sidak’s test). For total errors committed (Figure 3F) there was again a social condition × stage interaction effect (*F*_*1,20*_ = 4.8, *p* = 0.04): on average, as expected, SR mice committed more errors at CDR than CD (predicted LS-mean difference = 1.83, 95% CI = −0.80 to 4.47, *p* = 0.20), whereas, on average, SI mice committed fewer errors at CDR than CD (predicted LS-mean difference = −1.63, 95% CI = --4.41 to 1.15, *p* = 0.31).

### Effects of adolescent social isolation on orbital cortex glutamatergic synaptic proteins

**Figure 4:**
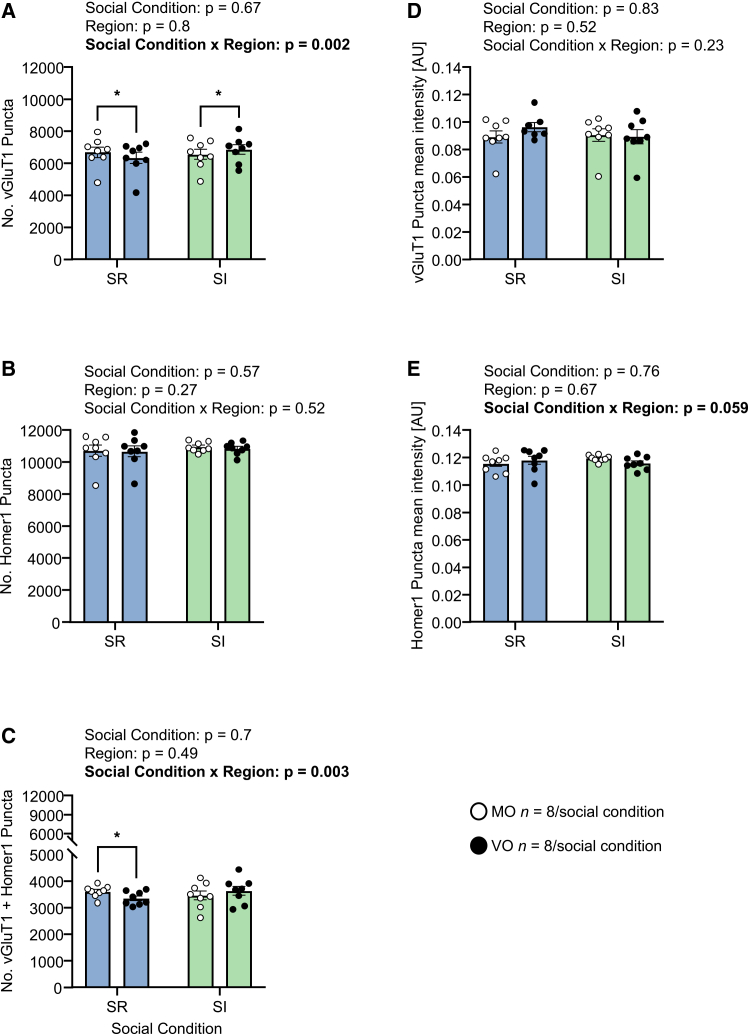
Effects of adolescent social isolation on maturation of orbital cortex glutamate synaptic proteins (A) Number of vGluT1 puncta according to social condition and OC subregion. (B) Number of Homer1 puncta according to social condition and OC subregion. (C) Number of colocalized vGluT1 + Homer1 puncta according to social condition and OC subregion. In (A–C), statistical analysis was conducted using two-way ANOVA. In the case of a significant social condition x OC subregion interaction effect, post hoc pairwise comparisons were conducted using Sidak’s test: ∗*p* < 0.05. (D) vGluT1 puncta mean intensity according to social condition and OC subregion. (E) Homer1 puncta mean intensity according to social condition and OC subregion. In (D and E), statistical analysis was conducted using either two-way ANOVA or, in the case of missing within-subject values, linear mixed-effect models. In (A–E), the data are individual values and group mean ± SEM. In all cases, outliers were identified using the ROUT method (Q = 1%) and removed: (A) no outliers; (B) no outliers; (C) no outliers; (D) 0–1 per social condition x region: total = 1/32; (E) no outliers.

In CON and SI littermates of the mice tested in terms of CSLT behavior, for the number of vGluT1 puncta per OC subregion (Figure 4A), there was a social condition x OC subregion interaction effect (*F*_*1,14*_ = 14.86, *p* = 0.002): in SR mice there were more vGluT1 puncta in MO than VO (mean difference = 349, 95% CI = 49 to 650, *p* = 0.023 in Sidak’s test), whereas in SI mice the opposite was the case (mean difference = −305, 95% CI = −606 to −5, *p* = 0.046). For the number of Homer1 puncta (Figure 4B), there was no consistent difference due to either social condition or OC subregion. For synaptic puncta (Figure 4C), reflecting the vGluT1 puncta differences, there was a social condition x OC subregion interaction effect (*F*_*1,14*_ = 13.15, *p* = 0.003): SR mice had more synaptic puncta in MO than VO (mean difference = 251, 95% CI = 46 to 457, *p* = 0.017 in Sidak’s test) whereas in SI mice the number of synaptic puncta was similar in MO and VO (mean difference = −169, 95% CI = −374 to 36, *p* = 0.11). Concerning mean puncta intensity, for vGluT1, there was no effect of social condition or OC subregion (Figure 4D); for Homer1, there was a social condition x OC subregion interaction effect (*F*_*1,14*_ = 4.23, *p* = 0.059; Figure 4E), and no post hoc comparison yielded significance.

In Expt. 1 there was a clear increase in total trials/total errors at CDR versus CD in socially reared mice aged 8 weeks, and it was unexpected that this was not replicated in the current experiment, i.e., in SR mice. Despite this, it was the case that social isolation resulted in a reduction in CDR trials/errors relative to CD, indicating that one or more of the neurobehavioral processes that typically mediate the specific challenge of reversal learning were disrupted by SI. There was no evidence that this was related to SI-induced changes in vGluT1 or Homer1 levels in the OC, as assessed in matched littermates of the mice studied behaviorally.

### Experiment 3: Effects of adolescent social isolation on complex sensory learning with differing task demands

In Experiment 1, week 6 mice made fewer errors at CDR than week 7 mice. In Experiment 2, despite the relatively weak increase in total trials and errors at CDR relative to CD in SR mice, we obtained evidence that SI from week 5 to testing at week 8 attenuated the increases in total trials and errors at CDR. While this could indicate that SI inhibits the development of adult-level reversal learning processes such as perseveration or learned non-reward at adolescence, it could also be the case that CDR is easier for SI mice because of reduced memory of CD learning on the previous day. To investigate this, we introduced a version of the CSLT in which SD, CD, and CDR were presented either on consecutive, separate days according to our standard protocol, or within one session on the same day. We then conducted a 2 social condition (SR, SI) x 2 task condition (one stage/day, all stages/day) experiment (Figure S1). The sample size was increased by 2 mice per group to increase power with respect to detecting a significant social condition x task condition interaction.

**Figure 5:**
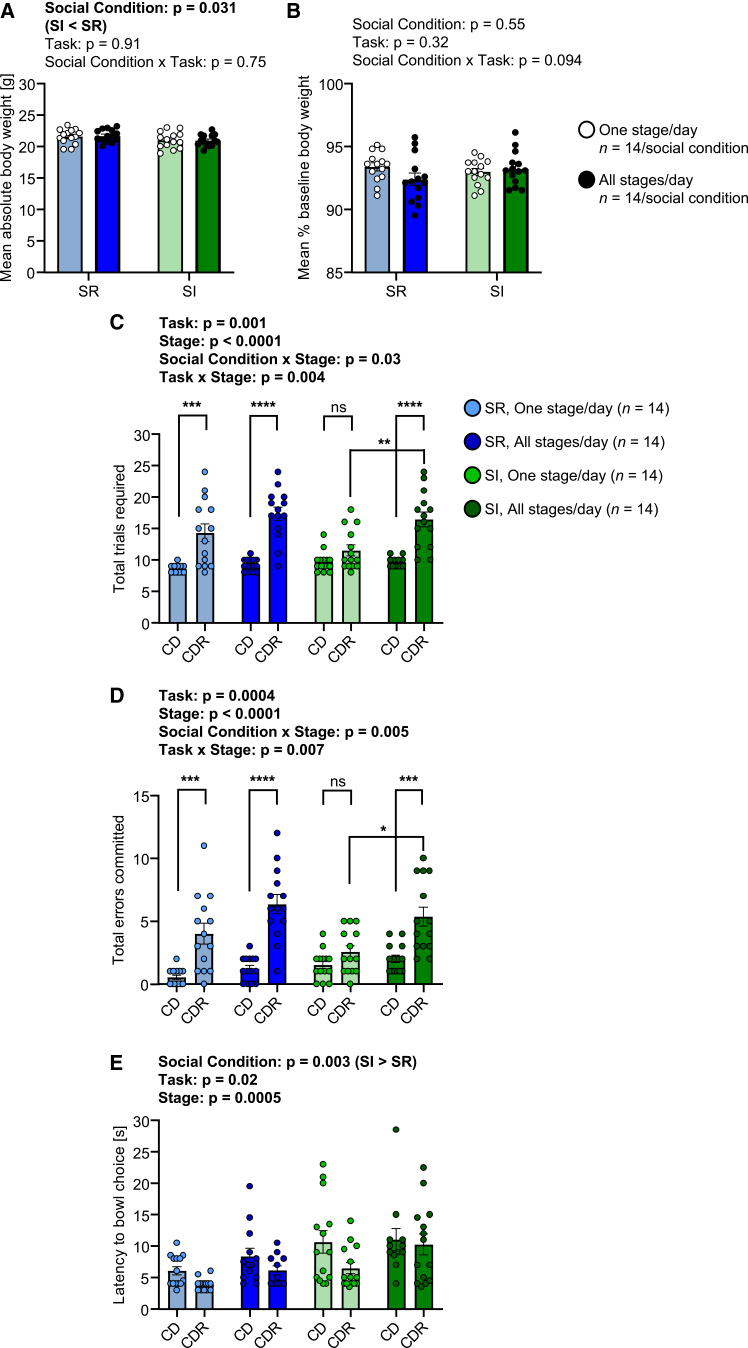
Effects of adolescent social isolation on behavior in the complex sensory learning task with differing task demands (A) Mean absolute body weight per mouse across 3 days of testing (i.e., one stage per day on separate days) and 1 day of testing (i.e., all stages on the same day). (B) Mean body weight per mouse as a percentage of baseline, where the latter was obtained immediately prior to the onset of food restriction for training and testing. In (A and B), statistical analysis was conducted using two-way ANOVA. (C) Total trials required to learning criterion according to social condition, task and stage. (D) Total errors committed to learning criterion according to social condition, task and stage. (E) Median latency to bowl choice according to social condition, task and stage. In (C–E), the data are given for stages CD and CDR specifically. Statistical analysis was conducted using linear mixed-effect models. In the case of a significant social condition x stage or task × stage interaction effect, post hoc pairwise comparisons were conducted using Tukey’s test: ∗∗∗*p* < 0.001, ∗∗∗∗*p* < 0.0001. In (A–E), the data are individual values and group mean ± SEM. In all cases, outliers were identified using the ROUT method (Q = 1%) and removed: (A) no outliers; (B) no outliers; (C) 0–2 per social condition x stage x task: total = 3/112; (D) 0–1 per social condition x stage x task: total = 1/112; (E) 0–3 per social condition x stage x task: total = 6/112.

Mice were mildly food restricted to 90–95% BBW during training and testing. For BW during testing (Figure 5A), SI mice were mildly but consistently lighter than SR mice (social condition main effect: *F*_*1,52*_ = 4.89, *p* = 0.031), in the absence of a task effect. For % BBW across test stages (Figure 5B), there was no effect of social condition or task condition, indicating consistent levels of food restriction. For required number of training sessions, there was a social condition × task interaction effect (*F*_*1,48*_ = 4.1, *p* = 0.048): SR-One stage/day mice required a relatively high mean of 3.8 days, SR-All stages/day mice required a mean of 3.0 days, SI-One stage/day and SI-All stages/day mice both required a mean of 3.4 days. There was no evidence that this chance difference in required training sessions between the 2 groups of SR mice was associated with any behavioral difference at testing. For total trials required (Figure S3A), there was a task × stage interaction effect (*F*_*2,101*_ = 5.31, *p* = 0.006). For total errors committed (Figure S3B), there was again a task × stage interaction effect (*F*_*2,103*_ = 6.15, *p* = 0.003). Furthermore, there was a social condition × stage interaction effect (*F*_*2,103*_ = 4.15, *p* = 0.018). This reflected, in part, a decrease in errors from SD to CD in SR mice (One stage/day *p* = 0.02, All stages/day *p* = 0.0008) and not in SI mice (One stage/day *p* = 0.56, All stages/day *p* = 0.94). It also reflected the absence of an increase in errors at CDR relative to CD in SI-One stage/day mice specifically. For bowl choice latency (Figure S3C), SI mice had longer latencies than SR mice (social condition main effect: *F*_*1,52*_ = 11.45, *p* = 0.001). There was also a task × stage interaction effect (*F*_*2,95*_ = 5.1, *p* = 0.008), with One stage/day mice taking a relatively long time to make a choice at SD compared with CD stages relative to All stages/day mice.

Given that the focus of this experiment was to investigate the effects of social isolation on reversal learning under short-versus long-term memory conditions, a planned comparison that included the stages CD and CDR specifically was conducted. For total trials required (Figure 5C), there was now a social condition × stage interaction effect (*F*_*1,49*_ = 4.9, *p* = 0.03): more trials were required at CDR versus CD in the case of the groups SR-One stage/day (predicted LS-mean difference = 5.73, 95% CI = 1.94 to 9.52, *p* = 0.0004 in Tukey’s test), SR-All stages/day (8.07, 4.36 to 11.78, *p* < 0.0001) and SI-All stages/day (6.75, 2.88 to 10.62, *p* < 0.0001), whereas this was not the case for SI-One stage/day mice (1.79, −1.92 to 5.50, *p* = 0.79); furthermore, more CDR trials were required by SI-All stages/day than by SI-One stage/day mice (4.93, 1.21 to 8.65, *p* = 0.002). For total errors committed (Figure 5D), there was also a social condition × stage interaction effect (*F*_*1,51*_ = 8.49, *p* = 0.005): more errors were committed at CDR versus CD in the case of the groups SR-One stage/day (3.49, 1.15 to 5.73, *p* = 0.0004), SR-All stages/day (5.14, 2.90 to 7.38, *p* < 0.0001) and SI-All stages/day (3.36, 1.12 to 5.60, *p* = 0.0004), whereas this was not the case for SI-One stage/day mice (1.07, −1.17 to 3.31, *p* = 0.79); furthermore, more CDR errors were made by SI-All stages/day than by SI-One stage/day mice (2.79, 0.41 to 5.16, *p* = 0.01). For bowl choice latency (Figure 5E), SI mice had longer latencies than SR mice (social condition main effect: *F*_*1,51*_ = 9.98, *p* = 0.003). Furthermore, All stages/day mice had longer latencies than One stage/day mice (task main effect: *F*_*1,51*_ = 5.5, *p* = 0.02), and mice required less time to make a choice at CDR than at CD (*F*_*1,47*_ = 13.86, *p* = 0.0005).

Socially reared mice, i.e., those experiencing social interactions across weeks 5–8, demonstrated the expected increases in trials and errors at the stage CDR versus stage CD at week 8, and did so regardless of whether CDR testing took place on the day after CD testing, the typical, one-stage/day condition used in this study, or immediately after CD testing in the all-stages/day condition implemented in this experiment specifically. Socially isolated mice at week 8 behaved similarly to SR mice in the all-stage/day condition, but did not demonstrate the expected increases in trials and errors at CDR versus CD in the one-stage/day condition. These findings indicate that reversal-specific processes such as perseveration and learned non-reward were intact in SI mice, and that impairment in long-term memory processes was responsible for their ease at reversal learning under the one-stage/day condition specifically.

## Discussion

The current iterative experiments provide novel insights into the development of and social isolation effects on complex discriminative learning and memory abilities in male mice between adolescence and adulthood. Furthermore, they provide concurrent evidence for the orbital cortex, a region known to be involved in the regulation of certain of these behavioral processes, in terms of glutamatergic synaptic maturation and social isolation effects thereon. As such, this study makes an important contribution to understanding the development of the processes involved in executive learning-memory and identifying those that are particularly susceptible to disruption by social isolation.

Whilst the study task used is based on the foraging ID/ED attentional set-shifting task established for rodents,^12^^,^^13^^,^^14^ we refer to it as complex sensory learning: in order to render the complete task solvable within 1-week training and 1-week testing, including by developing mice, only the early stages of the ID/ED task were included. Another feature that distinguished our CSL task from the typical ID/ED task was that only one stage was tested per day, thereby ensuring that any influence of the previous stage on current stage behavior would involve long-term (24-h) memory, or lack thereof, whereas standard ID/ED tasks make demands on flexible working memory. The main processes involved at each of the CSLT stages were: visual stimulus-reward discriminative learning-memory (SD); recalling the memory of this association in the presence of somatosensory distractor stimuli (CD); reversal learning of the memories of visual stimulus-reward/non-reward (CDR); novel visual stimulus-reward learning-memory of attentional set (IDS).

In Experiment 1, across all ages, mice required a similar number of trials/errors for SD, and then, also regardless of age, at CD required fewer trials/errors to recall the memory of this visual association despite the introduction of somatosensory distractor stimuli. When the associations between bead colors and reward were reversed (CDR), mice required more trials/errors than they did at CD, consistent with the dual requirements of having to unlearn the memory of the SD/CD bead color-reward association and learn that the non-rewarded bead color was now predictive of reward. This finding is consistent with a previous study of C57BL/6 adult mice,^13^ and provides the first evidence that such a reversal effect pertains 24 h after learning took place. Continuing to choose a previously rewarded, now unrewarded, stimulus until the acquired discriminative association memory is unlearned is perseveration, and its extent will depend primarily on memory processes and motivations to obtain reward/avoid non-reward.^6^ Continuing to avoid a previously unrewarded and now rewarded stimulus is learned non-reward, and its extent will depend primarily on memory processes and motivations to avoid non-reward/obtain reward.^5^ In addition, at CDR, mice aged 6 weeks made fewer errors than mice aged 7 weeks, strongly suggesting that this postnatal week constituted a critical period in the development of one or more of the processes underlying perseveration and/or learned non-reward. That the increase in errors was specific to week 7 suggests that mice can adjust to the maturation of reversal-inhibiting processes, at least to some extent, based on the experience they obtain in their home environment with changing response-outcome contingencies. Another possibility is that other executive functions, for example, sustained attention, develop in parallel across weeks 7–8, and lead to reductions in perseveration and learned non-reward.

This age effect was specific to CDR, with all age groups displaying a similar and efficient level of attentional set in terms of requiring relatively few trials/errors at IDS. Previous studies of young rodents (PND 26–60) also report that the number of trials/errors was similar at CD and IDS; in these studies, the formation of attentional set was confirmed by the increase in trials/errors from IDS to extra-dimensional shift (EDS).^11^^,^^27^^,^^38^

Given the evidence that reversal learning-memory performance changed across the age period investigated, we focused on the orbital cortex (OC), for which there is evidence for primate (marmoset)^8^ and rodent (rat, ^5^; mouse, ^35^) that excitotoxic lesioning in adulthood leads to impaired complex sensory reversal learning in the respective ID/ED tasks in which learning and reversal are typically tested within the same session. In rodents, these studies have focused on ventral, medial, and lateral OC. Using the glutamate neuron pre-synaptic protein vGluT1 and the post-synaptic neuronal density scaffolding protein Homer1, and in particular their punctal colocalization, a littermate of each of the behaviorally tested mice was perfused-fixed at the time point at which behavioral testing was completed, to enable the study of OC glutamate synapses in the absence of effects of CSL testing. For vGluT1, there was no clear evidence for age-related changes, whilst for Homer1, there was an increase in the number of puncta between age 5 weeks, when the number of puncta was lowest, and age 8 weeks, when the number of puncta was highest. In addition, the mean puncta intensity decreased between age 6 weeks and 8–12 weeks. Homer1 protein regulates glutamatergic synapses and dendritic spine morphogenesis.^39^ Despite the age-dependent differences in Homer1, there was no corresponding effect of age on the number of synaptic (vGluT1 + Homer1) puncta. Across all ages, there were more synaptic puncta in MO than in VO. Integrating the evidence for the dynamic status of CD reversal learning-memory between ages 6 and 7 weeks and the absence of any change in the number of OC vGluT1-Homer1 synapses at these ages, the current findings suggest that OC synapse maturation does not contribute majorly to the development of adult levels of reversal learning-memory between weeks 6 and 7, albeit the Homer1 changes might contribute in this regard via altered post-synaptic processing. Interestingly, OC lesioning (mainly lateral OC and to some extent ventral OC) in rats had opposite effects on perseveration and learned non-reward, which were decreased and increased, respectively, when CD and CDR were tested within a single daily session.^5^

Moving to the social isolation (SI) experiments, the evidence for the dynamic status of reversal learning-memory performance at ages 6 to 7 weeks informed the decisions to initiate SI at week 5 and to conduct CSLT testing at week 8, thereby encompassing this developmental period. Experiment 2 was the first experiment in which we investigated SI effects by comparing SI mice against a control group (SR) that was equivalent to the 8-week-old mice in Expt. 1, both in terms of CSLT behavior and OC glutamatergic synaptic proteins. Socially reared mice, specifically, required more trials at SD than at CD, consistent with Expt. 1, but SR mice required relatively few trials/errors at CDR versus CD compared with both Expt. 1 and published studies (e.g.,^13^). Relative to SR mice, at the SD stage, SI mice required fewer trials combined with a longer latency to bowl choice. This combination of findings could indicate an SI-induced increase in the exploration of the novel visual stimulus beads, leading to an increased ability to discriminate between bead colors and their respective associations with reward. Adolescent mice are, in the absence of any manipulation, more attracted by novelty than are adults,^40^ and, in addition, increased novelty exploration in SI mice and rats has been reported using various tasks, including novel object recognition.^41^^,^^42^^,^^43^^,^^44^ Another possible explanation for the group difference at SD is that SI increased sensitivity to non-reward, related to increased anxiety.^45^ Whilst SR mice displayed an attenuated reversal effect compared with Expt. 1. In SI mice, the reversal effect was non-existent, with even fewer trials being required at CDR than at CD. Restricting the analysis to CD and CDR stages only, it is apparent that SI mice required a relatively large number of CD trials; this is the stage at which the novel dimension of texture was introduced, so that again increased novelty exploration, this time of the distractor dimension, could have contributed to the SI effect observed. Powell et al. (2015) reported in rats that SI led to more CD trials than in SR subjects.^30^ Concerning effects of adolescent-onset SI on OC glutamatergic neuron synapses, for pre-synaptic vGluT1, there was a small but consistent shift in the relative number of medial OC versus ventral OC puncta, with MO > VO in SR mice and VO > MO in SI mice. Whilst there was no SI effect on post-synaptic Homer1 puncta levels, the effect of SI on vGluT1 meant that whereas SR mice had more synaptic puncta in MO than VO, this was not the case in SI mice. Social isolation from PND 31 to PND 60 led to increased dendritic spines and increased expression of the post-synaptic density protein 95 in the VO of adult mice, and this co-occurred with less goal-directed and more habitual operant responding.^46^

Based on the finding that weeks 6–7 constitute a developmental period of change in the processes underlying reversal learning of visual stimulus-reward/non-reward memories in mice (Expt. 1), and the finding of an unexpected absence of a clear reversal learning-memory effect in SR mice at week 8 (Expt. 2), we conducted a replication experiment to re-investigate the effects of adolescent SI on reversal learning-memory. Furthermore, we aimed to ascertain whether any observed SI effect was due to changes in the emotional processes underlying perseveration or learned non-reward per se, or rather to changes in the long-term memory processing of the visual stimulus-reward association. Therefore, in addition to SR and SI groups that were tested on one CSLT stage per day, such that the strength of the 24-h memory of the association could affect reversal behavior, we included SR and SI groups that were tested successively on all three stages – SD, CD, CDR – within one session. Firstly, in SR-One stage/day mice, the substantial increase in required trials/errors at CDR observed in Expt. 1 was observed again; indeed, the absolute values for trials and errors at CD and CDR were similar to those obtained for 8-week-old mice in Expt 1. It is noteworthy that Expts 1 and 3 were conducted by one and the same experimenter and Expt. 2 by a different experimenter, suggesting that inter-experimenter differences can, despite following the same protocol, introduce inter-experiment variability in tasks where human-animal interactions are continuous. In SR-All stages/day mice, the reversal effect was similar but even more pronounced, consistent with greater perseverative and/or learned non-reward effects when discrimination reversal followed directly after its acquisition. We consider this to be due to the recently formed CD memory being online, i.e., working, and resulting in the processes of perseveration and learned non-reward inhibiting CDR. Such an interpretation is in line with working memory theory, which suggests that it constitutes a process in which recently learned information is utilized actively to influence ongoing decision making.^2^ In SI-One stage/day mice, there was clearly no reversal effect, whilst in SI-All stages/day mice, the reversal effect was comparable to that in SR mice. In addition, whereas SR mice made fewer errors at CD than SD, consistent with efficient learning and memory recall of the visual stimulus that was correct at both stages, this was not the case for SI mice. Socially isolated mice had longer latencies to bowl choice than SR mice across all stages and regardless of task type, suggesting that they would be exposed to the discriminative stimuli for longer before making a stimulus choice.

These data are clearly consistent with adolescent SI disrupting long-term memory consolidation and/or recall for the visual-stimulus discriminative learning that occurred 24 h previously, whereas the working memory of a recently acquired visual discrimination is unaffected by SI. Furthermore, integrating the findings of this experiment and Expt. 1, it is possible that the weak reversal effect observed in mice at age 6 weeks is due to immature long-term memory processes relating to visual discriminative learning, and then that SI prevents/delays the maturation of these long-term memory processes. However, testing this hypothesis would necessitate conducting an additional experiment similar to Expt. 1 but incorporating both one-stage/day and all-stage/day conditions, with the prediction that the difference at weeks 6 and 7 would be specific to the former condition. The absence of this experiment is a limitation of this study and requires the integration of findings from Expts. 1 and 3 should be done with caution. The most relevant comparative data that are available for the effects of SI on reversal long-term memory are provided by experiments conducted with the Morris water maze: in line with the current findings, one rat study found that SI improved 24-h reversal learning^47^; however, another rat study observed that SI led to impaired 24-h reversal learning.^48^ Regarding SI effects on CDR with the typical, all stages per day protocol, the two relevant studies, both in rats, report that SI rats were impaired at CDR,^29^^,^^30^ for which there was no evidence in the current mouse Expt. 3.

Therefore, this study provides evidence that adult-level long-term memory of visual stimulus-reward/non-reward associations develops in adolescence in mice, and that adolescent social isolation impairs this development by altering long-term memory function specifically. As such, it provides novel insights into the processes via which social interactions/relationships, and paucity thereof, can regulate executive functioning. The current findings indicate that these long-term memory processes are not modulated by orbital cortex glutamatergic neuron synapses, and future studies can apply this interesting mouse model to investigate the neurobiological basis of the clear environment-behaviour inter-relationships identified.

### Limitations of the study

With respect to the limitations of the study, as noted above, in a future study, it would be important to repeat and expand on Expt 1, to both assess the replicability of the age effect on reversal learning and to determine whether the evidence for easier reversal learning is specific to less-efficient long-term memory or also applies to short-term memory. Given that the effects of age and social isolation were on long-term reversal memory of visual stimuli, it will be important to assess para(hippocampal) neurobiology, including glutamate neuron synapses, in future studies. The current study was restricted to males, and this should not mean that future studies do the same, with comparison of males and females being of interest, particularly from a translational viewpoint and sex differences in executive function disorders.

## Resource availability

### Lead contact

Further information and requests for resources and reagents should be directed to and will be fulfilled by the lead contact, Christopher R. Pryce (christopher.pryce@bli.uzh.ch).

### Materials availability

The study did not generate new unique reagents.

### Data and code availability

• All data in this article will be shared by the lead contact upon request.
• This article does not report original code.
• Any additional information required to reanalyze the data reported in this article is available from the lead contact upon request.

## Acknowledgments

We are grateful to Bozana Heil, Agnieszka Mojek, and Alex Osei for animal care, to Hannes Sigrist for technical support, and the Center for Microscopy and Image Analysis, University of Zurich, for assistance and support during confocal microscopy image acquisition. The research was funded by the University Research Priority Program (URPP), Adaptive Brain Circuits in Development and Learning (AdaBD), of the 10.13039/501100006447University of Zurich, Zurich, Switzerland.

## Author contributions

S.W. designed the study, acquired, analyzed and interpreted data (Expt. 1–3, behavior and neuroanatomy) and drafted the article. A.C. acquired, analyzed and interpreted data (Expt. 2, behavior), and drafted the article. G.P. acquired and analyzed neuroanatomy data, provided input for the design of the MATLAB script for neuroanatomy data analysis, and commented on the article. A.Ö.A. designed and wrote the MATLAB script for neuroanatomy data analysis, and commented on the article. T.K. interpreted data and commented on the article. C.R.P. conceived and designed the study, interpreted data, and drafted and edited the article.

## Declaration of interests

The authors declare no competing interests.

## STAR★Methods

### Key resources table

**Table:** 

REAGENT or RESOURCE	SOURCE	IDENTIFIER
**Antibodies**		

Guinea pig polyclonal anti-vGluT1	Synaptic Systems	Cat# 135304; AB_887878
Rabbit polyclonal anti-Homer1	Synaptic Systems	Cat# 160002; AB_2120990
Goat anti-guinea pig DyLight 650	Invitrogen, Thermo Fisher Scientific	Cat# SA5-10097; AB_2556677
Goat anti-rabbit Alexa Fluor 488	Invitrogen, Thermo Fisher Scientific	Cat# A11008; AB_143165

**Experimental models: Organisms/strains**		

Mouse (*Mus musculus*), C57BL/6JRj, male	Own breeding	–

**Software and algorithms**		

Fiji	ImageJ	https://imagej.net/ij/
Matlab	MathWorks	https://www.mathworks.com/products/matlab.html
Prism	GraphPad	https://www.graphpad.com/

**Other**		

Attentional Set Shifting (IDED) Chamber	Maze Engineers	https://maze.conductscience.com/portfolio/attentional-set-shifting-ided-chamber/
Aura Cacia, Pure Essential Oil, Lemon, 0.5 fl oz (15 ml)	iHerb	https://ch.iherb.com/pr/aura-cacia-pure-essential-oil-lemon-0-5-fl-oz-15-ml/6307
Coloured plastic beads	Hama the original beads	https://www.hamabeads.com/
Sucrose Pellets	Rodent Purified Diet, Bio-Serv	Dustless Precision Pellets, 20 mg, Chocolate Flavor

### Experimental model and study participant details

#### Animals and housing

All experiments were conducted with male C57BL/6 (BL/6) mice, bred in-house (breeding stock from Janvier, Le Genest-Saint-Isle, France). To minimize litter effect, a maximum of two male pups from any breeding pair were included per experiment. Pups were weaned at age 20-22 (mean 21) days and caged with 1, or occasionally 2, male littermates until age 26-32 days. From age 27-33 days until the end of the experiment, mice were housed in two social conditions: they either remained with their littermates, which was the control condition (social rearing, SR), or the littermates were separated and socially isolated (SI). All mice were kept in type 2 L cages (33 x 21 x 14 cm; Tecniplast, Italy) that contained sawdust, a sleeping igloo with two tissues for nest building, and crinkles (SAFE^R^ crinklets natural, SAFE complete care competence) for nest building and physical enrichment. For breeding and experimental mice, cages were maintained in an individually ventilated caging system with temperature and humidity kept at 21-23°C and 50-60%, respectively. The light-cycle was reversed with lights off from 7 AM to 7 PM. Mice were provided with autoclaved water and food croquettes (Complete pellet, Kliba Nafag, Granovit AG, Switzerland). Behavioural training and testing were conducted in an experimental room within the animal facility during the dark phase between 8 AM to 6 PM. Experiments were conducted under an animal experiment licence authorized by the Veterinary Office of the Canton Zurich (ZH-174/2021). Mouse well-being was monitored in terms of body weight, physical appearance and home cage behaviour, with such controls conducted on several days per week and daily during periods of food restriction.

#### Experimental design

Three iterative experiments were conducted in this study (Figure S1). Experiment 1 investigated species-typical development of complex sensory learning (CSL) and maturation of orbital cortex (OC) from adolescence to adulthood. Informed by Expt. 1, Experiment 2 investigated the effects of social isolation (SI) within this period on CSL development and OC maturation. Experiment 3 also investigated the effects of SI and, furthermore, added a version of the CSL task that allowed for effects of short- versus long-term memory on task performance, and SI effects thereon, to be assessed. In Expt. 1, the development of CSL was studied in a 2-week period per mouse, with mice aged weeks 5-6, 6-7, 7-8, 8-9 or 11-12 weeks (*n* = 12 per age group) in this period. Then, in naive same-sex littermates of the mice studied behaviourally, species-typical maturation of OC glutamate synapses was studied in terms of immunofluorescence staining, using mice aged 5, 6, 7, 8 or 12 weeks at perfusion-fixation (*n* = 8 per age group: the age 5 weeks was included additionally to the ages studied in terms of CSL and the age 9 weeks was not included). In Expt. 2, the effects of SI on CSL and OC synapses were investigated at age 7-8 weeks. Mice were either socially isolated from age week 5 (SI) or remained in their littermate pairs (SR). They then either underwent CSL training-testing at age 7-8 weeks (*n* = 12 per group) or remained behaviourally naive and were perfused-fixed for OC glutamate synapse immunofluorescence at age 8 weeks (*n* = 8 per group). In Expt. 3, the study of SI on CSL was replicated using a larger per group sample size (*n* = 14 per group) and including an additional task condition that allowed the effect of interval duration between successive CSL test stages, and potential interaction with SI, to be investigated.

### Method details

#### Complex sensory learning

##### Apparatus and materials

The apparatus for the complex sensory learning task (CSLT) comprised a grey PVC chamber (30.5 x 20.5 x 17.8 cm, L x W x H; Attentional Set Shifting (IDED) Chamber, Maze Engineers) divided into one starting compartment and two stimulus compartments. The starting compartment could be closed off from the stimulus compartments by a manually operated vertical divider out of grey PVC. The stimulus compartments were separated by a transparent Plexiglas divider. Ceramic bowls (Ø = 7.4 cm, H = 3.5 cm, Maze Engineers) that served as discriminative stimuli and contained sucrose reward, could be placed one per stimulus compartment. Two transparent Plexiglas lids containing ventilation holes could be placed on top of the starting and stimulus compartments to prevent mice from jumping out of the chamber. The chamber was placed on a table surface at a height of 72 cm. Lighting was provided by red light at the ceiling and low-level white light placed behind the chamber so that the 2 stimulus compartments were illuminated equally at 41 lux. The chamber and bowls were wiped with 70% ethanol between successive mice. For Expt. 3, which included longer test sessions than in Expt. 1 and 2, a water bottle was introduced so that its spout protruded through a hole in the centre of the end wall of the start compartment.

The sensory stimulus dimensions used were visual and somatosensory. The visual stimulus dimension was coloured plastic beads (Ø = 5 mm; Hama the original beads, https://www.hamabeads.com/) placed within the ceramic bowls (20 g per bowl), and the somatosensory stimulus dimension was smooth and rough textures of sandpaper or cardboard (JUMBO, Switzerland, https://www.jumbo.ch/de) wrapped inside and outside the bowls. The ceramic bowls and the beads were scented with lemon odour to mask any odour from the sucrose pellet (Dustless Precision Pellets, 20 mg, Chocolate Flavor, Rodent Purified Diet, Bio-Serv). Once a week, lemon oil (0.1 ml, Aura Cacia, Pure Essential Oil, Maze Engineers or iHerb) was pipetted onto filter paper which was then placed in the bowls, and the beads were placed on top. The filter paper was removed on the first day of training/testing, and overnight the bowls were stored in sealed plastic boxes to preserve the lemon odour across training/testing days.

##### Timeline

Each mouse proceeded through the consecutive periods of weaning (mean age postnatal day (PND) 21), familiarisation (mean age PNDs 23-29), standby and food restriction (variable duration and age at onset, respectively, depending on age at onset of training/testing), either prior to and during the 2-week training/testing period in the case of mice tested behaviourally, or during the littermate’s training/testing period in the case of mice studied in terms of neuroanatomy.

##### Weaning

At postnatal day (PND) 19-20, just prior to weaning, mice were given 20 sucrose pellets distributed in the home cage; the exposure to the pellets in the presence of the parents was expected to decrease any subsequent neophobic response. Mice were weaned at PND 20-22 (mean PND 21) and caged with 1, or occasionally 2, male littermates.

##### Familiarisation

In the familiarisation period mice were handled and introduced stepwise to the context and the stimuli used for CSLT training-testing. Thus, at age 4 weeks (typically PNDs 23-29, in some cases 1-2 days earlier or later) mice were weighed and handled daily for 5 min on each of 5 consecutive days. Furthermore, familiarisation steps were conducted day-specifically: Familiarisation day 1, 15 sucrose pellets per mouse were added to the home cage. Day 2, mice were ear marked for identification. Day 3, a lemon odour-treated ceramic bowl containing 15 sucrose pellets per mouse and 1 food croquette was introduced to the home cage, to habituate mice to entering and feeding in the bowls used in the CSLT. Day 4, if the pellets were eaten the bowl was removed, and if the pellets were still in the bowl it remained in the home cage. The home cage was transferred to the experimental room and remained there for 3-5 h of habituation. Day 5, the home cage was transferred to the experimental room for 0.5-1 h of habituation. The mice were then placed in and habituated to the CSLT apparatus. This included all mice per home cage, regardless of whether they were allocated to be investigated in terms of behaviour or neuroanatomy. All mice per home cage were transferred together to the starting compartment where home cage sawdust was sprinkled, and allowed to explore for 1 min. The experimenter sat behind the starting compartment and thereby allowed the mice to habituate to their presence. Two lemon odour-treated bowls, containing one sucrose pellet each, were placed one each in the corner of the stimulus compartments: the divider was manually removed so that the mice had access to and entered the stimulus compartments, and they were allowed to explore for 15 min. If eaten, sucrose pellets were replenished, to further encourage exploration and interaction with the bowls. The mice were then guided back to the starting compartment, the divider was inserted, and the mice transferred to the home cage.

##### Standby

Following familiarisation i.e. from age 5 weeks, mice entered “standby” which continued until they reached the study age period to which they were allocated and they commenced CSLT training and testing. Therefore, for example, in Expt. 1, mice allocated to age 5-6 weeks had no standby and mice allocated to age 8-9 weeks had 3 weeks standby. The onset of standby was the time point at which mice were allocated to groups, using a pseudo-randomized process, so that sample sizes for age groups (Expt. 1) and SR and SI groups (Expt. 2 and 3), as well as for behaviour or neuroanatomy, increased at a similar rate across the experiment. Mice were handled for 2 min per week during standby.

##### CSLT training and testing

Each CSLT training and testing had a maximum duration of 5 days, Monday-Friday, in consecutive weeks. On the Friday before onset of training, mice were handled for 5 min and weighed, and this body weight (BW) represented the baseline BW (i.e., 100% BBW). During CSLT training and testing, mice were mildly food restricted to 90-95% BBW; this was except for mice in the 5-6 weeks group which were restricted to only 95-100% BBW to allow for their increased growth relative to older age groups. To determine the amount of daily food to provide to study mice during CSLT training-testing, in another cohort of mice (*n* = 10), daily food consumption was measured during weeks 5-12, and the mean age-specific consumption (Table S1) was the amount given to study mice. To ensure mice were at the correct % BBW from the start of training, food restriction began 3 days before. On the Sunday before onset of training, a lemon odour-treated bowl with 15 sucrose pellets per mouse was placed in the home cage. Mice remained under daily food restriction throughout CSLT training-testing and were then returned to *ad libitum* feeding until euthanasia. Mice allocated to neuroanatomy also underwent food restriction so that they were also comparable to their CSLT littermates in this regard. In addition, during the littermates’ CSLT training-testing, they were placed in an unfamiliar home cage for approximately 30 min per day to mimic the experience of a context other than the home cage.

Training, or conditioning, and testing were conducted in the CSLT apparatus (Figure S2). For each training and testing trial, the mouse was transferred to the starting compartment for approximately 15 s whilst the lemon odour-treated stimulus bowls were prepared out of sight of the mouse. The divider was then manually removed so that the mouse had access to and entered the stimulus compartments, which was the onset of the bowl choice phase. As soon as the trial was completed, the mouse was guided back to the starting compartment, the divider was inserted, and the mouse began the waiting phase for the next trial. Training comprised 4 stages which differed in terms of the amount of sawdust placed in the stimulus bowls, such that mice could learn that digging in the sawdust could result in retrieval of a sucrose pellet. Stage 1 consisted of two empty ceramic bowls each containing one sucrose pellet. In stage 2, both bowls were half-filled with sawdust and the sucrose pellet was placed on top of the sawdust. In stage 3, both bowls were filled completely with sawdust and the sucrose pellet was located at the midline of the sawdust, and in stage 4 the pellet was underneath all the sawdust on the floor of each bowl. Each daily session consisted of 12 training trials maximum. If the mouse ate both pellets completely within 120 s, the trial was designated as “correct”; otherwise, the trial was “incorrect”. At each stage, 3 correct trials were required to pass. When a training stage was completed within a session, the mouse proceeded immediately to the next stage; if not, the number of correct trials was reset to 0 and the same training stage was resumed in the next day’s session. Training was completed when all 4 training stages were passed. The main readouts were number of sucrose pellets eaten per stage and total number of sessions needed to pass all stages.

In order that the CSLT could be trained and tested within a 2-week period, including by mice aged 5-6 weeks only, we modified the intra-dimensional/extra-dimensional attentional set-shifting tasks used in rats (e.g.,^12^) and mice (e.g.,^13^^,^^14^). Two lemon odour-treated bowls containing beads, one specific colour-shade per bowl (i.e., visual discriminatory-stimulus dimension), were wrapped with textured material, one specific texture per bowl (i.e., somatosensory distractor-stimulus dimension). The bowls were placed in the CSLT apparatus, one per stimulus compartment and out of sight of the mouse in the starting compartment. At each testing trial, a sucrose pellet was buried in one of the bowls and the mouse needed to acquire the predictive association that existed between one exemplar of the relevant sensory stimulus dimension (i.e., visual) and the sucrose reinforcement. For all mice, the visual-somatosensory stimulus combination and left-right positioning of the bowls across trials followed a pre-set and pseudo-randomised schedule. Testing comprised 4 stages (Figure S2). Stage 1 was simple discrimination (SD): one bowl contained white beads and the other dark-blue beads, and both bowls had a smooth, ceramic texture given that no somatosensory stimuli were applied. One colour-shade was reinforced, and this was counterbalanced across mice within each group. Stage 2 was compound discrimination (CD): two bowls were covered with fine sandpaper and contained either white beads or dark-blue beads, and two with coarse sandpaper and contained either white or dark-blue beads. Per trial, 1 fine and 1 coarse bowl were presented, and 1 contained white and 1 dark-blue beads, and the colour-shade reinforced at SD was relevant and texture was irrelevant, i.e. formation of an attentional set “visual stimulus” was reinforced. Stage 3 was CD reversal (CDR): the previously incorrect colour-shade was now correct and vice versa, while texture remained present and irrelevant. Reversal learning was therefore reinforced, and mouse choice behaviour could be influenced by perseveration (continuing to choose the previously correct, now incorrect, stimulus) and learned non-reward (continuing to avoid the previously incorrect, now correct, stimulus). Stage 4 was intra-dimensional shift (IDS): two novel bead colour-shades were introduced - light-blue and brown - as were two new textures - ridged versus smooth cardboard. Attentional set visual stimulus was still correct, and the ability to apply this to novel stimuli was tested, and the reinforced colour-shade was light-blue if it was white at SD/CD and brown if it was dark-blue at SD/CD.

Each daily testing session had a maximum of 34 trials per stage, and a maximum duration of 60 min in Expt. 1 and 2 and 120 min in Expt. 3. These 34 trials comprised 4 initial “free” trials and 30 “closed” trials. In the free trials, at each stage, each combination of colour-shade, texture and right-left compartment position was presented once, and only the correct bowl contained a sucrose pellet. This aimed to familiarise mice with the discriminatory stimuli and did not contribute to the test performance score. Per free trial, the mouse had 120 s maximum to explore and make a bowl choice: an incorrect choice could be made without consequence and the mouse could subsequently explore the correct bowl; as soon as a correct choice was made, the incorrect bowl was removed, and the mouse could eat the sucrose pellet. A free trial was terminated after either no bowl choice (i.e. scored as an incorrect choice) or a correct choice. After the 4 free trials, the subsequent 30 closed trials began. Per closed trial, the mouse had 120 s maximum to explore both bowls and make a bowl choice. In the case of a correct choice, the incorrect bowl was removed immediately and the mouse had 30 s to investigate and find and consume the sucrose pellet (positive reinforcement); in the case of an incorrect choice, the correct bowl was removed immediately and the mouse had 30 s to investigate the incorrect bowl (positive punishment). Bowl choice was defined as the mouse displaying digging behaviour i.e., moving beads in the bowl with its front paws and/or snout. Only contacting or sniffing the bowl, and/or nibbling on a texture, was not a bowl choice. If the mouse made neither a correct nor an incorrect choice within 120 s, this was an omission trial. Overall, there were few omission trials and those that did occur did so primarily during “free” trials; any omissions during experimental trials occurred at stage SD. Omission trials were counted as error trials. The criterion for learning a discrimination and passing a stage was 8 correct choices in 8–10 consecutive trials (Figure S2). If a mouse did not pass a stage within the 30 closed trials, it began the next day’s session on this same stage and the number of consecutive correct trials was reset to 0. Testing was completed when all 4 test stages were passed. In Expts. 1 and 2, mice were tested on one stage only per daily session, to ensure that hunger/motivation was similar across stages and to increase the relevance of long-term (24 h) memory to between-stage effects. In the case of Expt. 3, because we were also interested in the effect of 24-hour memory on CD reversal learning, for both SR and SI, in addition to a cohort that was tested on one stage per day there was a cohort that was tested on all stages on the same day. To make this possible, the IDS stage was omitted in Expt. 3. In each experiment, the main CSLT test readouts were the total trials required and total errors committed to reach learning criterion at each test stage, and also the median latency to make a bowl choice.

##### Euthanasia

On the Monday following completion of testing, mice that had been in behavioural experiments were euthanized using carbon dioxide asphyxiation followed by bilateral pneumothorax. Littermates for neuroanatomical study simultaneously underwent perfusion-fixation.

#### Orbital cortex synaptic protein imaging

##### Perfusion-fixation and brain tissue preparation

Mice were deeply anaesthetized with sodium pentobarbital (150 mg/kg) injected intra-peritoneally. Mice were perfused for 1 min with 10 ml of 1x PBS (pH 7.4, RT) and then perfused-fixed for 3 min with 30 ml of 4% paraformaldehyde (PFA, 4°C) in 0.1 M sodium phosphate buffer (pH 7.4). After perfusion-fixation, the brains were removed from the skull and post-fixed in 4% PFA for 2 h, at 4°C. For subsequent cryoprotection, the brains were rinsed twice in 1x PBS and then went through a ramping scale of sucrose solutions in 1x PBS: 10%, 15%, 30%, each for 24 h at 4°C. The brains were then embedded in cryoprotective medium (Tissue-TEK O.C.T. Compound, Sakura Finetek) in a cryomould, frozen on powdered dry ice, and stored at -80°C until sectioning.

##### Histology

Coronal brain sections from bregma level +2.70 mm to +1.70 mm, and therefore including orbital cortex (OC), were cut at 40 μm using a freezing microtome (Histocom AG, Switzerland). Sections were collected in 1x PBS (pH 7.4, RT) and transferred into a 96-well plate (Tissue culture plate, 96 well, Sarstedt AG & Co. KG), with each well filled with 200 μl of tissue collection solution (TCS at RT: 250 ml Glycerin, 300 ml Ethylene glycol, 250 ml 0.2 M PO_4_, 250 ml dH_2_O). The 96-well plates were stored at -20°C until immunofluorescence staining was conducted.

##### Immunofluorescence staining

For quantification of pre- and post-synaptic proteins, and of their colocalization as synaptic puncta, immunofluorescence co-staining was conducted. Vesicular glutamate transporter 1 (vGluT1) was used as pre-synaptic protein, and post-synaptic density scaffolding protein (Homer1) as post-synaptic protein. Per mouse, two coronal sections of the OC (bregma level +2.10/2.06 mm) were selected for immunofluorescence. In Expt. 1 (*n* = 5 groups x 8 brains x 2 sections/brain), immunofluorescence was conducted in two runs, and all age groups were included in a counterbalanced manner in each run. In Expt. 2 (*n* = 2 groups x 8 brains x 2 sections/brain), immunofluorescence was conducted in one run. For each staining run, a negative control (i.e. section near the region of interest) was taken from a control mouse (mouse aged 5 weeks for Expt. 1, and SR mouse for Expt. 2).

Pilot immunofluorescence staining demonstrated that optimal signal coverage for vGluT1 and Homer1 was obtained by incubating sections with the former in the absence of the latter for 24 h, followed by a period of incubation with both antibodies for a further 24 h. The immunofluorescence staining protocol spanned three consecutive days (days 1 to 3). The following staining steps were performed using 24-well plates (Tissue culture plate, 24 well, Falcon^R^), with each well containing the 2 sections from one mouse. For every washing step and incubation period, the 24-well plates were placed on a shaker (120 rpm) at RT. On day 1, to remove all tissue collection solution, the sections were placed in 1x PBS (pH 7.4) and washed for 1 h (3 x 20 min). They were then transferred to blocking solution (300 μl/well) of 20% normal goat serum (NGS) with 1x PBS for 1 h. The sections were then transferred to a solution (240 μl/well) of guinea pig polyclonal anti-vGluT1 (1:500, 135304, Synaptic Systems) in 0.3% Triton in 1x PBS and 10% NGS, for 24 h at 4°C. On day 2, a solution (10 μl/well) containing 0.3% Triton in 1x PBS, 10% NGS and rabbit polyclonal anti-Homer1 (1:20, 160002, Synaptic Systems) was added to the wells, giving a final anti-Homer1 concentration of 1:500. The sections were incubated for 24 h at 4°C. On day 3, unbound primary antibodies were removed by transferring the sections to 1x PBS and washing for 1 h (3 x 20 min). Sections were then returned to the well plates and incubated for 2 h with a solution (300 μl/well) containing the fluorophore-conjugated secondary antibodies, goat anti-guinea pig DyLight 650 (1:250, SA5-10097, Invitrogen, Thermo Fisher Scientific) and goat anti-rabbit Alexa Fluor 488 (1:250, A11008, Invitrogen, Thermo Fisher Scientific), in 0.3% Triton in 1x PBS and 10% NGS. This incubation and all following steps were performed in the dark. To remove unbound secondary antibodies, the sections were transferred to 1x PBS and washed for 1 h (3 x 20 min). The sections were mounted on microscope slides (Superfrost™ Plus Adhesion Microscope Slides, New Erie Scientific LLC, Epredia) with a solution containing 50% 1x PBS and 50% dH_2_O, air dried, dipped in dH_2_O, air dried again. For cell nucleus staining, DAPI Fluoroshield Mounting Medium (Fluoroshield™ with DAPI, Sigma-Aldrich, Co.) was used. Cover slips (24 x 50 mm, New Erie Scientific LLC, Epredia) were added, and slides were stored in a closed microscopy box maintained at 4°C until image acquisition.

##### Image acquisition

In Expt. 1 and Expt. 2, image acquisition was conducted 1-5 days after completion of immunofluorescence staining. For each of the medial and ventral orbital cortices (MO, VO), one image was acquired per hemisphere and section; with ROIs identified using a mouse brain atlas.^49^ For MO, ROI acquisition was conducted at layers 2/3 and for VO at layers 5/6, informed by the Allen Mouse Brain Atlas.^50^ Imaging was conducted using an automated inverse confocal laser scanning microscope (Leica SP8) running the LSX software. The Leica SP8 objective used was HCX PL APO 37°C CS2 63x/1.3 NA glycerol. With the hybrid (HyD) detectors in photon counting mode, the fluorescence channel DyLight 649 was used for vGluT1, with the HyD detector set at the range 646-800 nm and laser power at 10%, and the fluorescence channel ALEXA 488 was used for Homer1, with the HyD detector set at 500-562 nm and laser power at 8%. Due to a “sandwich effect” with the vGluT1 signal, specifically the well-impregnated surface (z-size = 8.70 μm) of the brain section that was in contact with the microscope slide was imaged, with a z-step size of 0.30 μm and 30 acquisition steps. By choosing 1024 x 1024 as format, scan speed of 400 Hz with a bidirectional scan direction X, zoom of 3, frame accumulation of 2 and a pinhole of 1 AU, ROI acquisition was at a final image size of 61.51 x 61.51 μm and pixel size of 60.13 x 60.13 nm. The z-step size of 0.30 μm was 2x that proposed by the Nyquist calculator such that we slightly under-sampled but were still within the valid range according to Nyquist sampling.

##### Synaptic puncta detection and colocalization analysis

To analyse synaptic puncta, image z-stacks from the two separate fluorescence channels were processed independently to determine colocalization levels. Intensity normalization was first applied to correct for variations in fluorescence intensity across different samples, ensuring consistency in downstream analysis. A top-hat filtering operation was used to enhance the contrast of puncta by selectively removing background fluorescence while preserving small, bright features of interest. Following this, Wiener filtering was applied to reduce noise and improve the clarity of puncta signals. Wiener filtering is an adaptive technique that minimizes the impact of noise by considering local variations in intensity and estimating the signal-to-noise ratio, leading to enhanced feature detection without introducing excessive smoothing.

Puncta were detected using a Laplacian of Gaussian (LoG) filter, a commonly used edge-detection method that enhances regions of high curvature, making it particularly effective for identifying punctate structures with Gaussian-like intensity profiles. The LoG filter first applies a Gaussian smoothing to suppress noise and small variations, followed by the Laplacian operator to highlight regions with rapid intensity changes, effectively pinpointing puncta-like structures. This approach ensures robust detection of puncta by accounting for their size and shape characteristics. After detection, median filtering was applied to further suppress residual noise and refine the detected structures. Quality thresholding was then used to exclude spurious detections by setting criteria based on puncta size and intensity, ensuring only biologically relevant puncta were retained for analysis.

Colocalization analysis was performed by estimating the volumetric overlap of puncta between the two channels. This was achieved by assessing the degree of overlap in the Z-dimension and computing the total overlapping area within the 2D image plane, using known pixel dimensions to derive accurate volumetric measurements. This approach enabled precise quantification of colocalization by integrating both lateral and axial spatial components, providing a comprehensive understanding of the spatial relationships between synaptic markers. The analysis yielded quantitative outputs such as puncta counts, colocalization volumes, and fluorescence intensities, which were subsequently used for statistical comparisons and interpretation of synaptic colocalization. For each output used, in the case of each mouse and ROI, the mean value of (2 hemispheres x 2 sections) was calculated and used for statistical analysis.

### Quantification and statistical analysis

#### Statistical analysis

The sample size in Expt. 1 was informed using ANOVA power analysis (GPower 3.1) with medium Cohen’s f effect size = 0.30, α = 0.05, power = 0.80, number of groups = 5, and number of measurements = 4, which yielded a required total sample size = 60, i.e. 12 mice per group. Given that there were outlier mice in Expt. 1 and Expt. 2, then the sample size that contributed to statistical analysis was sometimes n=10-11 per group rather than the aimed for n=12. Accordingly, the sample size was increased to n=14 per group in Expt. 3, thereby allowing for 2 outliers per group and for an expected n=12 per group in the statistical analysis. Both the behavioural and neuroanatomical data were analysed using GraphPad Prism (version: 9.5.1). The data sets were assessed for outliers using the ROUT method (Q = 1%), and all outlier values were excluded from statistical analysis. Data were analysed using one-, two- or three-way mixed model analysis of variance (ANOVA), or a generalised linear mixed-effects model (LMM) in the case of missing (outlier) data points for repeated measures. In Expt. 1, behavioural data were analysed by LMMs with fixed effects of age and stage and random effect of mouse subject, and neuroanatomical data with ANOVA with a between-subjects factor of age and within-subjects factor of OC subregion, or LMMs in the case of missing data points. In Expt. 2, behavioural data were analysed by LMMs with fixed effects of social condition and stage and random effect of mouse subject, and neuroanatomical data with a between-subjects factor of social condition and within-subjects factor of OC subregion. In Expt. 3, behavioural data were analysed with fixed effects of social condition and task condition and random effect of mouse subject. In the case of significant between-subjects interaction effects or within-subjects main effects post hoc testing was conducted using Tukey’s or Sidak’s multiple comparisons test as appropriate: the pairwise mean difference (predicted least-square mean difference in the case of LMM) and the 95% confidence interval for the difference between means, were calculated, together with the adjusted p value. Given that it is complex and rare to calculate effect size estimates for LMM analysis, and there are not well-established guidelines with respect to the relationship between values and effect size, effect size estimates were not included. All tests were 2-tailed and statistical significance was set at p ≤ 0.05. Where asterisks are used to indicate significant p values, the nomenclature is as follows: ∗ p < 0.05, ∗∗ p < 0.01, ∗∗∗ p < 0.001, ∗∗∗∗ p < 0.0001.
