# Supplementary material for: Development of visual-stimulus reversal learning-memory in mice is dependent on social interaction
Source: iScience. 2026 Jan 31;29(3):114864. doi: 10.1016/j.isci.2026.114864 (PMC12930062; doi:10.1016/j.isci.2026.114864)
Supplement: Document S1. Figures S1–S3 and Table S1 [file mmc1.pdf]

iScience, Volume *29*

Supplemental information

Development of visual-stimulus reversal learning-memory in mice is dependent on social interaction

Sarah Wicki, Annika Canziani, Giulia Poggi, Ali Özgür Argunşah, Theofanis Karayannis, and Christopher R. Pryce

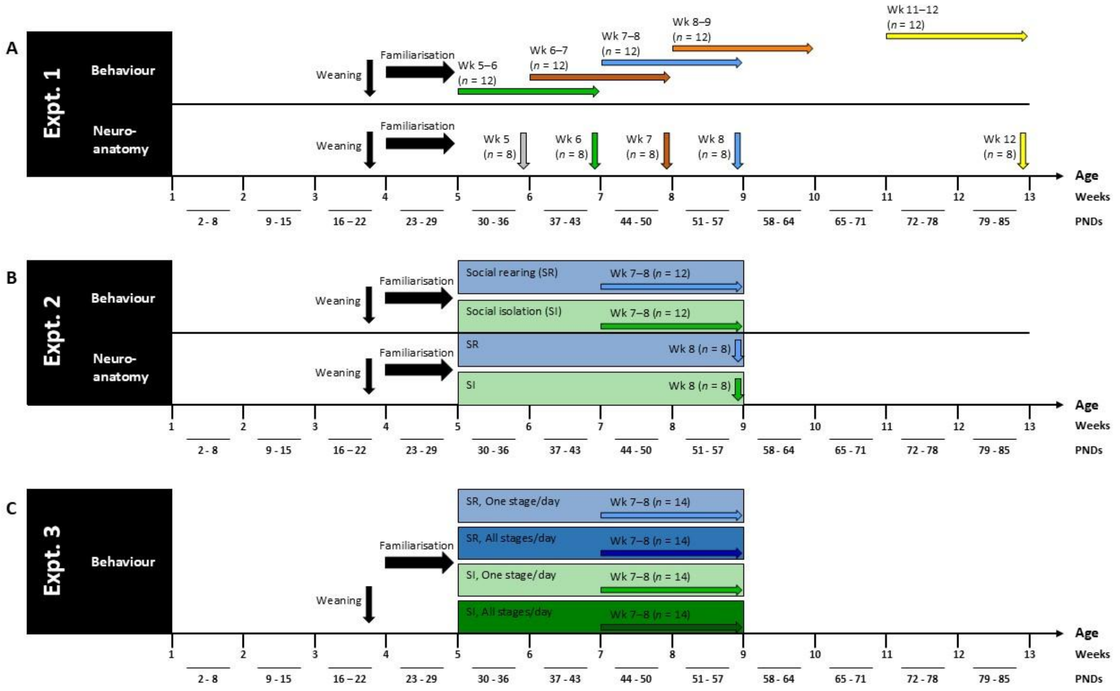

Figure S1. Overview of the designs of the three iterative experiments conducted. (A) Experiment 1 investigating species-typical development of complex sensory learning (CSL) and maturation of orbital cortex (OC) from adolescence to adulthood. **(B)** Experiment 2 investigating effects of social isolation (SI) from week 5 on CSL behaviour and OC glutamate synaptic proteins at week 8. **(C)** Experiment 3 investigating effects of SI from week 5 on CSL behaviour in the standard task of 1 stage per day and the modified task of all stages per day. For age in postnatal days (PNDs), there is a mean shift of 1 day between chronological age and experimental age: this is due to mice born at the weekends not entering the study until the Monday, so that study week 1 covers PNDs 2-8 rather than 1-7, and so on. For behavioural experiments, the horizontal arrows indicate the chronological age at which 1 week of training followed by 1 week of testing was conducted. For neuroanatomical experiments, the vertical arrows indicate the chronological age at which the mouse was perfused; this was at the end of week indicated.

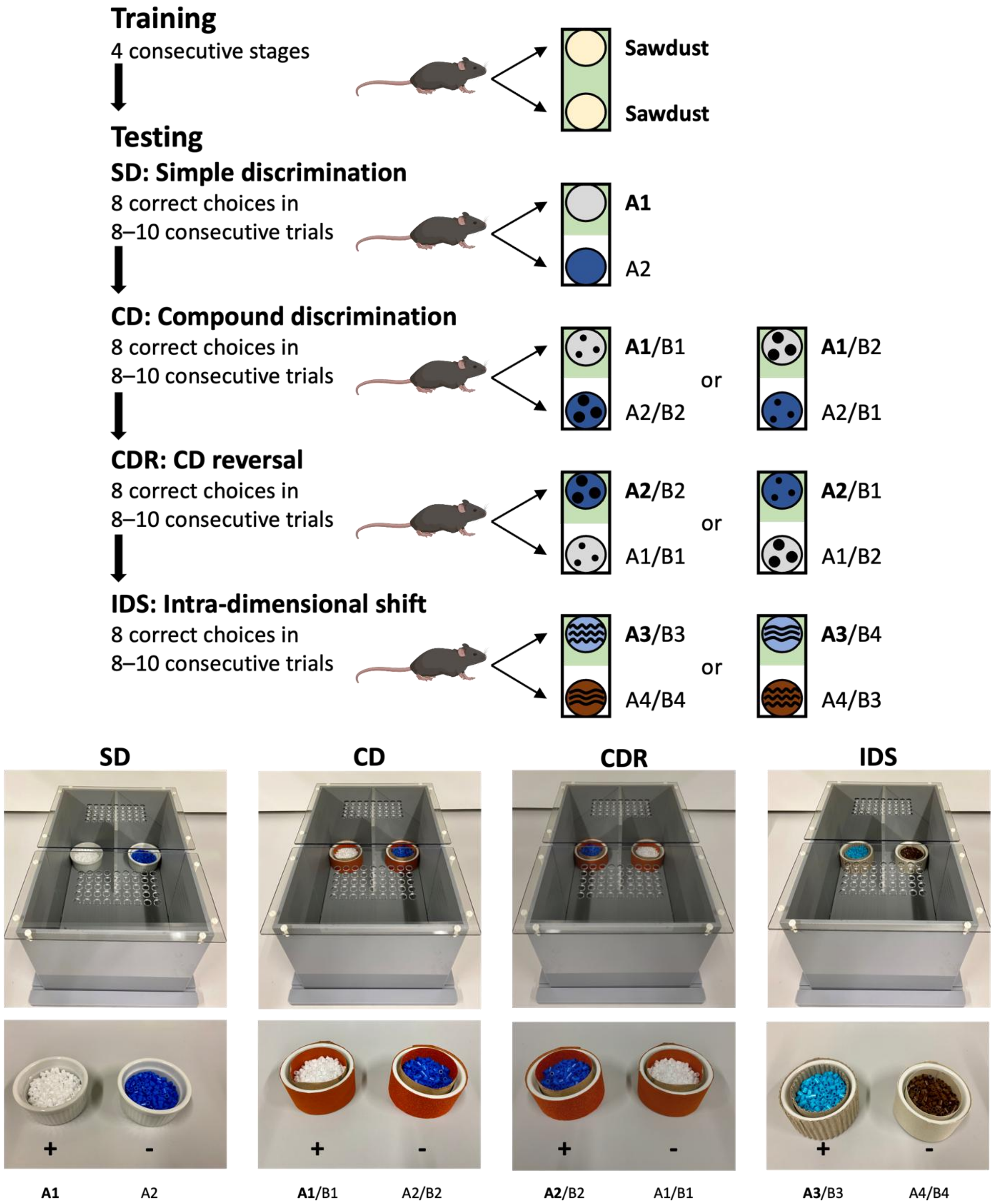

Visual stimulus dimension

A1: White beads
A2: Dark-blue beads
A3: Light-blue beads
A4: Brown beads

Somatosensory stimulus dimension

B1: Fine Sandpaper
B2: Coarse Sandpaper
B3: Ridged Cardboard
B4: Smooth Cardboard

Figure S2. Explanation of the complex sensory learning task. Mice were first trained to dig in sawdust to retrieve sucrose pellets. Trained mice then underwent the successive stages of the task: simple discrimination (SD), compound discrimination (CD), CD reversal (CDR) and intra-dimensional shift (IDS). In the task, mice needed to learn which specific colour-shade per bowl (i.e., visual discriminatory

stimulus dimension) was associated with the sucrose pellets, and also needed to learn to ignore the textured material in which the bowls were wrapped (i.e., somatosensory discriminatory stimulus dimension). One bowl was placed in each of the stimulus compartments. In 50% of mice, the correct visual stimulus was white beads (A1) at stages SD and CD, dark-blue beads (A2) at CDR, and light-blue beads (A3) at IDS; in the other 50% of mice, the correct visual stimulus was dark-blue beads (A2) at stages SD and CD, white beads (A1) at CDR, and brown beads (A4) at IDS. The sensory stimulus combinations and left-right positioning were pseudo-randomised across trials and for each stage were identical in all mice. Upper images were created with BioRender.com.

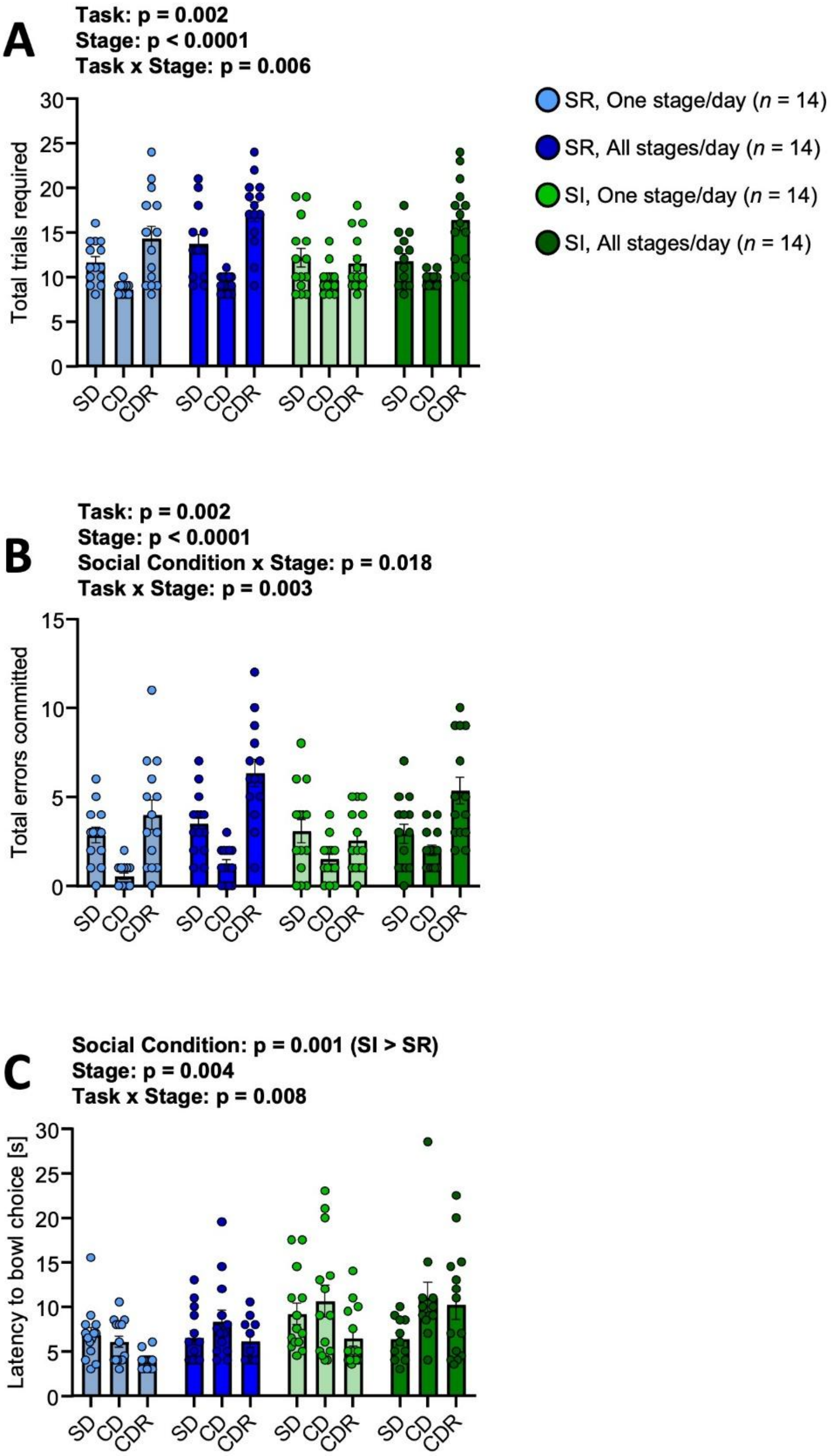

Figure S3. Effects of adolescent social isolation on behaviour in the complex sensory learning task with differing task demands.

(A) Total trials required to learning criterion according to social condition, task and stage. **(B)** Total errors committed to learning criterion according to social condition, task and stage. **(C)** Median latency to bowl choice according to social condition, task and stage. In **(A)-(C)**, statistical analysis was conducted using linear mixed-effect models. In the case of a significant social condition x stage or task

x stage interaction effect, post hoc pairwise comparisons were conducted using Tukey's test. In **(A)** to **(C)**, the data are individual values and group mean ± SEM. In all cases, outliers were identified using the ROUT test (Q = 1%) and removed: **(A)** 0-2 per social condition x stage x task, total = 3/168; **(B)** 0-1 per social condition x stage x task: total = 1/168; **(C)** 0-3 per social condition x stage x task: total = 9/168.

Table S1. Age-specific body weight and food intake used to inform main experiments.

Age in weeks, postnatal days	Body weight [g] Mean ± SD	Food intake per day [g] Mean ± SD
4, 22-28	12.9 ± 1.2	NA
5, 29-35	18.9 ± 1.1	3.3 ± 0.3
6, 36-42	21.8 ± 1.0	3.5 ± 0.3
7, 43-49	23.9 ± 0.9	3.5 ± 0.3
8, 50-56	25.0 ± 0.9	3.7 ± 0.3
9, 57-63	26.0 ± 0.9	3.6 ± 0.2
10, 64-70	26.8 ± 1.0	3.6 ± 0.1
11, 71-77	27.2 ± 1.1	3.4 ± 0.2
12, 78-84	27.7 ± 1.1	3.4 ± 0.1

n = 10
